# Impaired autophagy triggered by HDAC9 in mesenchymal stem cells accelerates bone mass loss

**DOI:** 10.1186/s13287-020-01785-6

**Published:** 2020-07-03

**Authors:** Liqiang Zhang, Meng Qi, Ji Chen, Jiangdong Zhao, Liya Li, Jiachen Hu, Yan Jin, Wenjia Liu

**Affiliations:** 1 National & Local Joint Engineering Research Center of Biodiagnosis and Biotherapy, Precision Medicine Institute, The Second Affiliated Hospital of Xi’an Jiaotong University, Xi’an, 710004 China; 2 State Key Laboratory of Military Stomatology & National Clinical Research Center for Oral Diseases & Shaanxi International Joint Research Center for Oral Diseases, Center for Tissue Engineering, School of Stomatology, The Fourth Military Medical University, No. 145 West Changle Road, Xi’an, 710032 Shaanxi China; 3 Xi’an Institute of Tissue Engineering and Regenerative Medicine, Xi’an, 710032 Shaanxi China; 4 The Key Laboratory of Aerospace Medicine, Ministry of Education, The Fourth Military Medical University, Xi’an, 710032 Shaanxi China

**Keywords:** Bone mass loss, HDACs, BMMSCs, Autophagy, Lineage differentiation

## Abstract

**Background:**

Bone mass loss in aging is linked with imbalanced lineage differentiation of bone marrow mesenchymal stem cells (BMMSCs). Recent studies have proved that histone deacetylases (HDACs) are regarded as key regulators of bone remodeling. However, HDACs involve in regulating BMMSC bio-behaviors remain elusive. Here, we investigated the ability of HDAC9 on modulation of autophagy and its significance in lineage differentiation of BMMSCs.

**Methods:**

The effects of HDAC9 on lineage differentiation of BMMSCs and autophagic signaling were assessed by various biochemical (western blot and ChIP assay), morphological (TEM and confocal microscopy), and micro-CT assays.

**Results:**

Sixteen-month mice manifested obvious bone mass loss and marrow fat increase, accompanied with decreased osteogenic differentiation and increased adipogenic differentiation of BMMSCs. Further, the expression of *HDAC9* elevated in bone and BMMSCs. Importantly, HDAC9 inhibitors recovered the lineage differentiation abnormality of 16-month BMMSCs and reduced p53 expression. Mechanistically, we revealed that HDAC9 regulated the autophagy of BMMSCs by controlling H3K9 acetylation in the promoters of the autophagic genes, *ATG7*, *BECN1*, and *LC3a/b*, which subsequently affected their lineage differentiation. Finally, *HDAC9* inhibition improved endogenous BMMSC properties and promoted the bone mass recovery of 16-month mice.

**Conclusions:**

Our data demonstrate that HDAC9 is a key regulator in a variety of bone mass by regulating autophagic activity in BMMSCs and thus a potential target of age-related bone loss treatment.

## Background

Osteoporosis is a common aged-related disease and is characterized by decrease bone mass and bone mineral density, leading to bone fragility and a higher risk of fractures [1, 2]. The increasing incidence of fracture in senile osteoporosis has become a heavy burden on health care worldwide, especially in China, which has a growing aging population. Researchers have discovered several risk factors associated with osteoporosis, including genetic and epigenetic factors, hormone imbalance, and stem cell senescence [2–4].

Bone marrow mesenchymal stem cells (BMMSCs) are a group of cell residual in the bone marrow. They have self-renewal capacity and multilineage differentiation potential. There is considerable data showing that BMMSCs play crucial roles in maintaining bone remodeling, reparation, and regeneration [5, 6]. Importantly, the number of BMMSCs declines and their lineage commitment shifts from osteoblasts to adipocytes with aging [7, 8] leading to an imbalance between bone mass and bone marrow fat. This imbalance is considered to be a hallmark of aged-related bone loss disorder, osteoporosis.

During senescence, mesenchymal stem cells (MSCs) undergo epigenetic and transcriptional changes, including decreased expression of stemness genes, *Oct4* and *Nanog* [9], and increased age-related genes, *p16* and *p53* [5, 10]. Some adverse factors that trigger MSC senescence have been identified, such as reactive oxygen species (ROS) accumulation, telomere shortening, and epigenetic effectors, including histone deacetylases (HDACs) and DNA methyltransferases (DNMTs) [11, 12]. However, the details of the epigenetic regulation network remain elusive and its roles in BMMSCs during aged-related bone loss remain to be established.

HDACs are important epigenetic regulators that control gene transcription by removing acetyl groups from lysine side chains in histones and other proteins [13, 14]. Mammalian HDACs are divided into four classes based on their structure and function. Class I HDACs consist of *HDAC1*–*3* and *HDAC8*, while class II HDACs include *HDAC4–7*, *HDAC9*, and *HDAC10*. Class III HDACs, also known as named sirtuins (*Sirt1–7*), differ from the other HDACs, as they are dependent on nicotinamide adenine dinucleotide. Class IV HDAC has only one member, *HDAC11*. HDAC family members have been shown to be involved in a wide range of aging-associated diseases, including muscle atrophy, loss of physical activity, and neurodegenerative diseases [13, 15, 16]. Recent evidence indicates that HDACs are key regulators on bone remodeling. For example, *HDAC3* and *HDAC8* supported bone formation [15], while *HDAC4*, *HDAC7*, and *HDAC9* promoted bone resorption [17–19]. However, whether and how HDACs regulate BMMSCs senescence remains unclear.

In this study, we report that HDAC9 plays an important role in maintaining the balance between osteogenesis and adipogenesis of BMMSCs during aged-related bone mass loss. Furthermore, we found that the downregulation of HDAC9 could partially reverse the differentiation of aging BMMSCs and bone loss by regulating autophagy both in vitro and in vivo. These results suggest that aged-related bone mass loss may be partially controlled by the HDAC9-meditated autophagy of BMMSCs.

## Methods

### Animals

All animal procedure, operations, and experiments were approved and performed, and experimental protocols were approved by the guidelines of the Animal Care Committee of the Fourth Military Medical University, Xi’an, Shaanxi, China. Two-month-old female C57BL/6J mice were purchased from the Animal Experimental Center of the Fourth Military Medical University. Sixteen-month-old female C57BL/6J mice were purchased from Changzhou Kaiwensi Laboratory Animal Center, Jiangsu, China. All mice were housed under specific pathogen-free conditions (22 °C, 50–55% humidity) on a 12-h light/12-h dark cycle with food and water easily accessible.

### Micro-computed tomography analysis

The mouse femora at mid-diaphysis were scanned with the GE micro-CT system (GE, USA). X-ray source was set at 80 kV and 80 μA microfocus. Three-dimensional images were reconstructed, and data analysis was performed with GEHC MicroView analysis software. The relevant bone morphometric parameters, including trabecular bone mineral density (BMD), trabecular volume relative to total volume (BV/TV), and cortical bone thickness (Ct.Th), were measured.

### Isolation and culture of C57B/L BMMSCs

Bone marrow-derived mesenchymal stem cells were harvested from the femora and tibiae of 2-month-old young C57BL/6J and 16-month-old aged C57BL/6J mice. Mice were sacrificed with cervical dislocation and sterilized with 75% alcohol. The femora and tibiae were separated, and attached muscle was stripped. After epiphyses were amputated and the bone marrow was exposed, primary BMMSCs were drawn out; cultured with basal medium containing α-MEM medium (Gibco, Grand Island, NY, USA), 20% FBS (Sijiqing, Hangzhou, China), 2 mM l-glutamine (Invitrogen, Carlsbad, CA), 100 U/mL penicillin, and 100 U/mL streptomycin (North China Pharmaceuticals Company, Shijiazhuang, China); and incubated at 5% CO_2_ at 37 °C. The medium was changed every 3 days. Cells were digested with 0.25% trypsin when confluence reached 90%. BMMSCs used for the majority of experiments were at passage 1 in this study.

### Isolation and culture of C57B/L myoblasts

Muscles were harvested from the hind limbs of 2-month-old young C57BL/6J and 16-month-old aged C57BL/6J mice. Firstly, the excessive connective tissues and fat were separated from muscle in cold sterile PBS. Then, muscles were cut into small pieces and enzymatically digested with 400 IU/mL collagenase II (Worthington) at 37 °C for 1 h. The digested slurry was sequentially passed through a 70-μm and then 30-μm cell strainer (BD Falcon). The filtrate was centrifuged at 1000*g*, and the pellets were suspended in myoblast growth medium (Ham’s F-10 medium with 10% FBS) and incubated at 5% CO_2_ at 37 °C. Briefly, the cell suspension was seeded for 15–30 min to allow quick adherence of fibroblasts, thus leaving a purer population of myoblasts in the supernatant, which was then transferred to another dish for subculturing. Myoblasts were digested with 0.25% trypsin when confluence reached 80%.

### Senescence-associated β-galactosidase staining

The femora were fixed in 4% paraformaldehyde, decalcified with 17% EDTA (pH 7.0), dehydrated with 30% sucrose, and embedded in OCT. Fifteen-micrometer-thick longitudinal sections were prepared and collected on slide for SAbetaGal staining. The β-galactosidase activity was assessed with a SAbetaGal staining kit (Cell Signaling). Briefly, the slices were washed twice with PBS and fixed with Fixative Solution of the SAbetaGal staining kit at room temperature for 10–15 min. Then, the samples were washed twice with PBS, covered with SAbetaGal staining solution, and incubated at 37 °C overnight. On the next day, the slides were washed with PBS three times and 80% glycerin was mounted on the samples. Then, coverslips were inverted onto slide and excess glycerin was removed. The SAbetaGal positive cells were observed under a microscope.

### Osteogenic and adipogenic differentiation assays

Young and aged BMMSCs were seeded at the density of 4.2 × 10^4^ cells/cm^2^ on 6-well or 12-well plastic plates and cultured with basal medium. When cell confluence reached 80%, cells were induced with osteogenic differentiation medium (100 nmol/L dexamethasone, 50 μg/mL ascorbic acid, and 5 mmol/L β-glycerophosphate) up to 1 week for western blot assay or 2 weeks for Alizarin Red staining assay, with the medium changed every 3 days. To assess osteogenic differentiation, cells were fixed with 4% paraformaldehyde and stained with 1% Alizarin Red. The expressions of Runx2 and ALP were detected by western blot.

Young and aged BMMSCs were cultured using the same method described above. When cell confluence reached 85%, cells were induced with adipogenic differentiation medium (0.5 mol/L 3-lisobutyl-1-methyxanthine, 200 μmol/L indomethacin, 1 μmol/L hydrocortisone, and 10 μg/mL insulin) up to 5 days for western blot or 7 days for Oil Red O staining, with the medium changed every 3 days. Lipid droplet formation in cells was detected by staining with Oil Red O solution. The expression of PPAR-γ was detected by western blot.

### Oil Red O staining

For cell Oil Red O staining, the BMMSCs were fixed with 4% paraformaldehyde for 15 min at room temperature and then stained with Oil Red O staining for 15 min at room temperature. The stained cells were washed twice with PBS and then observed under microscope. The images were analyzed with Image Pro Plus software.

### Alizarin Red staining

Alizarin Red staining was performed to determine mineralization after 14 days of osteogenic induction. BMMSCs were fixed with 60% isopropanol for 90 s and washed once with ddH_2_O. Then, the cells were stained with 1% Alizarin Red (Sigma Aldrich) for 3–5 min and washed twice with ddH_2_O. Quantitative parameters of the mineralized area were analyzed with Image J software.

### Alcian blue staining

Alcian blue staining was carried out to detect the chondrocytes in growth plates of the femora. Firstly, the samples of decalcified femora were cut into 10 μm thick and mounted on slides. Then, samples were stained with 0.1% Alcian blue staining solution containing acetic acid for 20 min at room temperature. Then, slides were washed twice with ddH_2_O and the positive areas were observed under microscope.

### Immunofluorescent staining

For cell immunofluorescence staining, cells were seeded on 3.5 cm confocal dish at the density of 3 × 10^4^. Cells were treated with or without the autophagy-flux inhibitor chloroquine (CQ) (50 μM) 3–5 h before staining. In sequence, cells were fixed with 4% paraformaldehyde at 4 °C for 10–15 min, washed with PBS, incubated with 0.5% Triton-100 at room temperature for 10 min, and blocked with PBS containing 1% BSA at room temperature for 40 min. Next, the samples were incubated with primary antibodies to LC3 (Cell Signaling Technology, 1274, 1:100), aggrecan (GeneTex, GTX54920, 1:100), collagen II (Abcam, ab34712, 1:100), OCN (Santa Cruz Biotechnology, sc-390877, 1:100), PPAR-γ (Abcam, 2435, 1:50), and TRAP (Santa Cruz Biotechnology, sc-30833, 1:100) overnight at 4 °C and subsequently incubated with fluorescent secondary antibodies, respectively. The positive cells were examined under a laser scanning confocal microscope (Olympus FluoViem FV 1000, Tokyo, Japan). Quantitative histomorphometric analysis was conducted with Image Pro Plus software.

### Transmission electron microscopy analysis

Cells were harvested and fixed in 4% glutaraldehyde in 0.1 M PB (pH 7.4) for 24 h, followed by 1% osmic acid for 2 h. After fixation, cells underwent osmosis by acetone and 812 resins. Then, samples were embedded with Epon 812 and kept in a thermostatic drying oven for 4 h at 36 °C, 6 h at 45 °C, and 12 h at 60 °C. Afterwards, embedding blocks were successively cut into semithin sections (1–2 μm) and ultrathin sections (50–100 nm). Then, samples were stained with uranyl acetate and lead citrate. Finally, images were captured with a transmission electron microscope (FEI, USA) at an accelerating voltage of 80–120 kV.

### qRT-PCR analysis

Total RNA was extracted from BMMSCs, myoblasts, bone marrow, and muscle by RNAiso plus (TaKaRa, Japan) according to the manufacturer’s instruction. Then, the mRNA was reversely transcribed into cDNA by PrimescriptTM RT master mix (TaKaRa, RR036A). Real-time PCR was performed with SYBR Premix Ex TaqTMII (TaKaRa) and detected by CFX96 Trademark Real-time PCR detection system (Bio-Rad, USA). The primers used in real-time PCR were listed in the Supplementary Table 1.

### Western blotting analysis

Total proteins were harvested from BMMSCs, bone marrow, and other organs with RIPA lysis buffer (Beyotime, China) and quantified by BCA assay. Next, the proteins were separated on sodium dodecyl sulfonate-polyacrylamide gels (SDS-PAGE), transferred to PVDF membranes (Millipore, Billerica, MA, USA), blocked in TBST containing 5% BSA, and incubated in first antibodies with Beclin1 (Cell Signaling, 3738, 1:1000), ATG7 (Cell Signaling Technology, 8558, 1:1000), LC3 (Cell Signaling Technology, 1274, 1:1000), p62 (Proteintech, 18420-1-AP, 1:1000), HDAC9 (Abcam, ab59718, 1:1000), H3K9ac (Abcam, ab10812, 1:1000), H3K18ac (Cell Signaling Technology, 9675, 1:1000), H4K16ac (Abcam, 13534, 1:1000), H3 (Cell Signaling Technology, 9715, 1:1000), p53 (Cell Signaling Technology, 2524, 1:1000), phospho-p53 Antibody (R&D systems, AF1043, 1:2500), Runx2 (Cell Signaling Technology, 2435, 1:1000), ALP (R&D systems, AF2910, 1:500), PPAR-γ (Abcam, 2435, 1:300), and GAPDH (Cwbiotech, CW0100, 1:4000), respectively. Then, the membranes incubated in secondary antibodies which coupled to peroxidase (Cwbiotech, China). Finally, the signals were detected by an enhanced chemiluminescence kit (7seapharmtech, China).

### HDAC inhibitor TSA and NaB treatment

BMMSCs were cultured in the medium with Trichostatin A (TSA, sigma) at 50, 100, and 200 (nmol/L) or sodium butyrate (NaB, sigma) at 50, 100, 200, and 400 (μmol/L). To assess the effects of TSA or NaB on inhibition of HDACs in cells, HDAC1–11 were detected by qRT-PCR. The expressions of HDAC9 and acetylated H3k9 were detected by western blotting. To examine the effects of HDACs on BMMSC differentiation, the cells were treated with or without NaB or TSA at their effective concentrations selected above.

### siRNA interference

Small interfering RNA (siRNA) targeting mice BECN1 (Ribobio, China) or HDAC9 (Santa Cruz, USA) were transfected into cells at a final concentration of 50 nM using riboFECT™ CP (Ribobio, China). siNC (Ribobio, China) was used as negative controls. All steps were performed according to the instruction in the riboFECT™ CP kit. The following experiments were performed according to the experiment designed. In detail, siRNA silencing HDAC9 was transfected into BMMSCs to investigate the effects of HDAC9 on osteogenic and adipogenic differentiation of BMMSCs and binding to the promoter of autophagy-related genes. In addition, aged BMMSCs were co-transfected with siHDAC9 and siBECN1 to investigate the hypothetical HDAC9-autophagy axis which may regulate BMMSC lineage differentiation.

### Chromatin immunoprecipitation

To confirm the interaction between HDAC9 and targeting genes, chromatin immunoprecipitation assays were performed according to the manufacturer’s protocol (Millipore, LSKMAGG02, USA). Antibodies against HDAC9 (Abcam, ab59718) and polyclonal anti-histone H3 (acetyl K9) (Abcam, ab10812) were incubated with randomly interrupted genome DNA samples. Normal rabbit IgG (Merck Millipore) was used as a negative control. Then, antibody-DNA complexes were captured by protein A/G magnetic beads. Finally, the precipitated DNA samples were detected by qRT-PCR, and the results were normalized to the input value. The primers designed according to the promoters of Atg7, BECN1, LC3a, and LC3b (Sangon biotech, China) are listed in Supplementary Table 2.

### shHDAC9 virus injection in vivo

The shRNA sequences for targeting HDAC9 were as follows: forward sequence—5′-CCGGCTGGGCACAAAATTCTGAACACTCGAGTGTTCAGAATTTTGTGCCCAGTTTTTG-3′; reverse sequence—5′-AATTCAAAAACTGGGCACAAAATTCTGAACACTCGAGTGTTCAGAATTTTGTGCCCAG-3′. The shHDAC9 lentivirus was packaged by co-transfecting shHDAC9 lentiviral vector with two packaging systems (pMD2.G and psPAX2) in 293T cells which were purchased from China Center for Type Culture Collection (CCTCC, GDC187), and the medium containing virus was collected and concentrated 48 h later. Detailedly, the medium was centrifuged at 800 rpm and 4 °C for 10 mins, and the supernatant was filtered by a 0.45-μm filter. Then, the filtrate was mixed with Lenti-X (TaKaRa, 631,231) and kept at 4 °C overnight. The next day, the mixture was centrifuged for 40 min at 1500*g* and the precipitate was resuspended with PBS whose volume is 1/100 of collected medium. The final virus titer was more than 1 × 10^8^/mL. To evaluate the effect of HDAC9 on bone mass/skeletal metabolism in senescence-induced bone loss, aged mice (*n* = 21) were randomly divided into three groups (7/each group), namely the control group, the shScr group, and the shHDAC9 group, respectively. Aged mice were anesthesized through intraperitoneal injection with 1% pentobarbital sodium and were administrated with 20 μL of lentivirus containing shHDAC9 or empty vector every 2 weeks for 1 month through intra-bone marrow injection in the distal femora. After a 1- or 2-month treatment, the mice from above three groups were sacrificed by cervical dislocation and the femora from each group were collected for micro-CT scanning, Oil Red O staining, and SAbetaGal staining. BMMSCs were harvested and cultured from the femora for lineage differentiation analysis and autophagy analysis as above methods.

### Statistics

The data were presented as the mean ± SD. Comparisons were made using *t* test and one-way ANOVA for experiments with more than two groups. All of the experiments were repeated more than three times, with representative experiments shown. The *P* values of less than 0.05 were considered significant.

## Results

### HDAC9 level increased in BMMSCs and bone marrow during aged-related bone mass loss

**Fig. 1:**
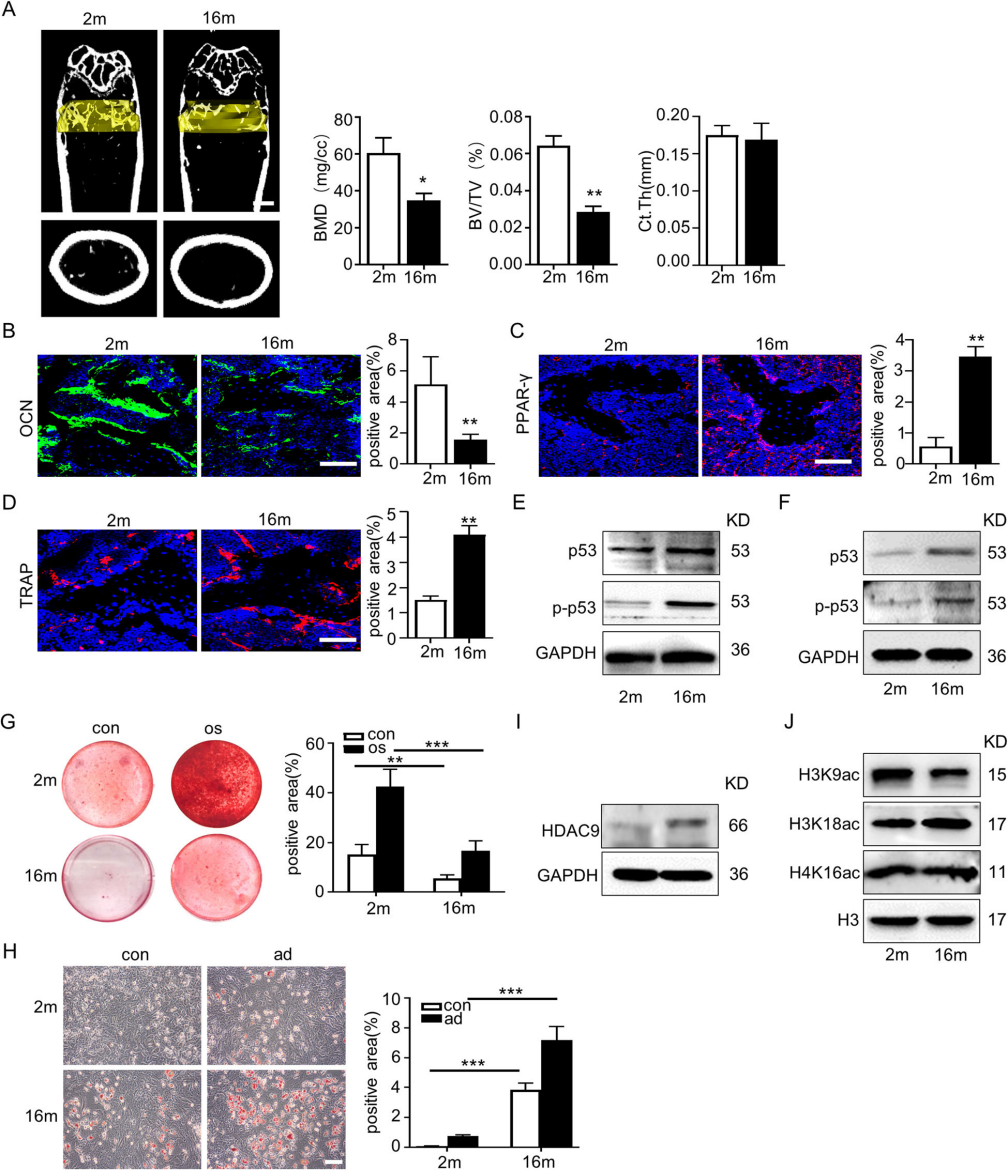
The increase of HDAC9 was associated with bone and fat imbalance in bone aging. **a** Micro-CT analyses of bone mass of trabecular and cortical bone thickness in the femora of 2-month-old (young) and 16-month-old (aged) mice. Bone mineral density (BMD), trabecular bone volume (BV/TV), and cortical bone thickness (Ct.Th) were performed. Scale bar = 1 mm. **b**–**d** Immunofluorescent staining of OCN (**b**), PPAR-γ (**c**), and TRAP (**d**) was performed in the bone marrow from young and aged mice, and the positive signals were quantitatively analyzed. Scale bar = 50 μm. **e** Expressions of the senescence-related proteins, p53 and p-p53, in the bone marrow from young and aged mice were examined by western blotting. **f** Expressions of the senescence-related proteins, p53 and p-p53, in BMMSCs from young and aged mice were examined by western blotting. **g** Alizarin Red staining was performed, and quantification of mineralized nodules was analyzed in young and aged BMMSCs. **h** Oil Red O staining was performed, and quantification of lipid droplet positive ratio areas was analyzed in young and aged BMMSCs. Scale bars = 100 μm. **i**, **j** Expression of HDAC9 (**i**) and acetylation sites of H3K, including H3K9, H3K18, and H4K16 (**j**), were examined by western blotting. The data are presented as the means ± SD of each independent experiment performed in triplicate. *P < 0.05, **P < 0.01, ***P < 0.001, unpaired two-tailed Student’s *t* test

Firstly, young mice (2-month-old) and aged mice (16-month-old) were compared. The micro-CT analysis showed that the aged mice presented typical aged-related bone mass loss, including decreased bone mineral density (BMD) and trabecular bone volume (BV/TV), whereas no cortical bone thickness difference was observed (Fig. 1a). Moreover, increased numbers of TRAP-positive osteoclasts and PPAR-γ-positive adipocytes and reduced expression of OCN were observed in the bone marrow of aged mice (Fig. 1b–d). Immunoblotting showed higher protein expression levels of senescence-related proteins p53 and p-p53, in aged mice compared with those in young mice (Fig. 1e). Given that HDACs have been implicated as key factors in the pathogenesis of age-related disorders and diseases, gene expressions of HDAC family members were measured in the bone marrow from young and aged mice. Only *HDAC9* expression remarkedly increased, while *HDAC5* and *HDAC7* expression levels reduced in aged mice. Meanwhile, there was no significant difference in the expression levels of *HDAC1–4*, *HDAC6*, *HDAC8*, *HDAC10*, or *HDAC11* in the bone marrow from young and aged mice (sFig. 1a).

To screen for changes in *HDAC* gene expression, BMMSCs were isolated from the femora of young and aged mice respectively and cultured in vitro. The senescence-associated proteins p53 and p-p53 were highly expressed in BMMSCs from aged mice compared to those from young mice (Fig. 1f). The aged BMMSCs possessed significantly impaired potential osteogenesis capacity and higher adipogenesis ability compared with young BMMSCs (Fig. 1g, h). RT-PCR result showed almost an approximately tenfold increase in the level of *HDAC9* mRNA and significant decrease in the level of HDAC5 mRNA of aged BMMSCs. Expression levels of the other HDACs were not significantly different between the two groups of cells (sFig. 1b). Based on the increased HDAC9 expression both in the bone marrow and BMMSCs of aged mice, we focused on the potential roles of HDAC9 in aged-related bone mass loss. We analyzed the level of HDAC9 protein and key histone acetylation sites, including H3K9, H3K14. and H3K18 in young and aged BMMSCs by western blotting. Significantly increased expression of HDAC9 (Fig. 1i) and decreased level of H3K9 acetylation (Fig. 1j) were observed in BMMSCs from aged mice compared with those from young mice. However, no differences were found in the acetylation level of H3K18 or H4K16. In the light of the importance of communication between bone and muscle, skeletal muscle and myoblasts were examined in young and aged mice. The increased expression of p53, p-p53, and HDAC9 and decreased acetylation level of H3K9 in aged BMMSCs were also observed in muscles and myoblasts from aged mice (sFig. 2a-f).

### Inhibition of HDAC9 rebalance lineage differentiation in aged BMMSCs

**Fig. 2:**
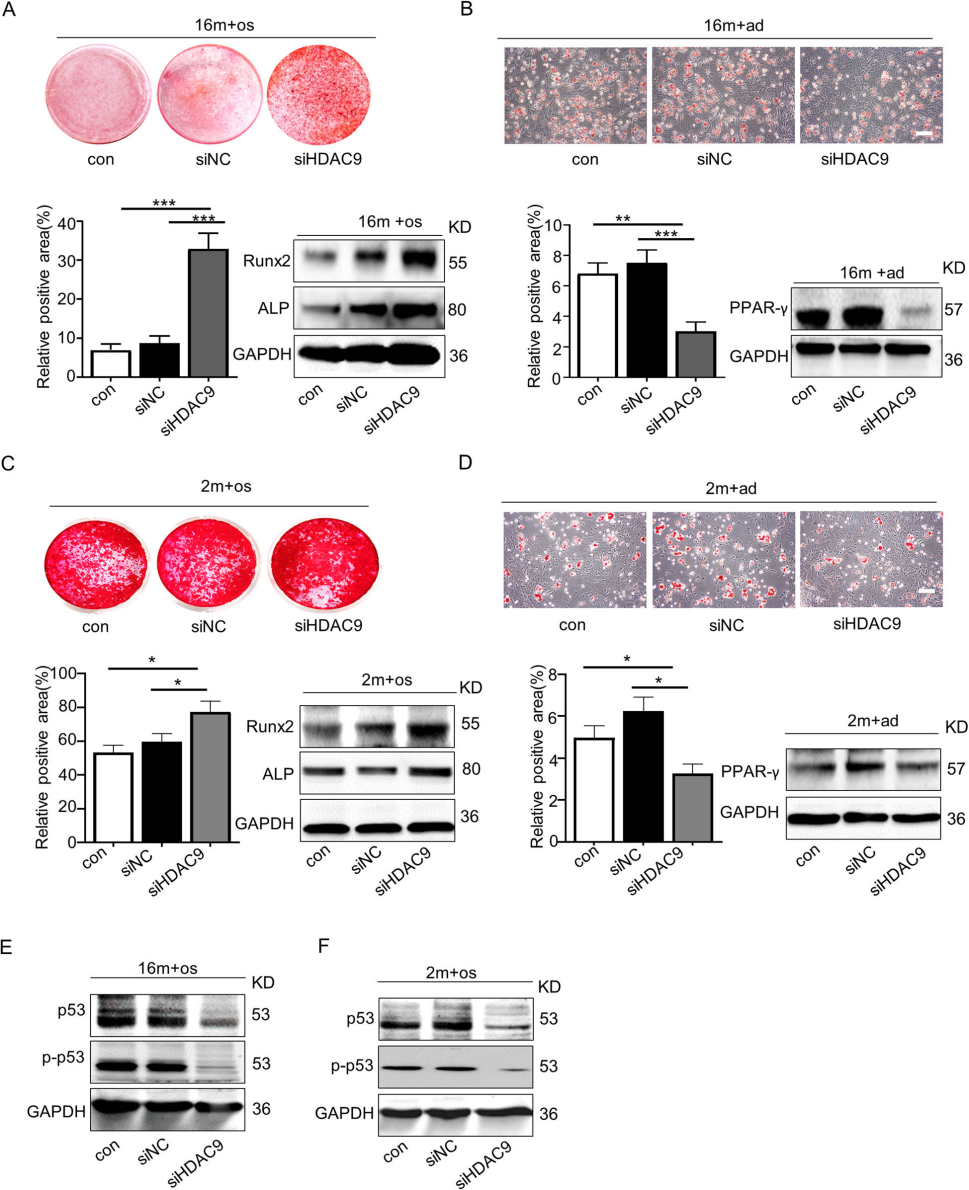
Downregulation of HDAC9 rescued lineage differentiation imbalance and ameliorated senescence in aged BMMSCs. **a** Alizarin Red staining was performed, and osteogenesis-related proteins were detected by western blotting in aged BMMSCs transfected with *HDAC9* siRNA. **b** Oil Red O staining was performed, and adipogenesis-related proteins were detected by western blotting in aged BMMSCs transfected with *HDAC9* siRNA. **c** Alizarin Red staining was performed, and osteogenic-related proteins were detected by western blotting in young BMMSCs and young BMMSCs transfected with *HDAC9* siRNA. **d** Oil Red O staining was performed, and adipogenic-related proteins were detected by western blotting in young BMMSCs and young BMMSCs transfected with HDAC9 siRNA. **e**, **f** Expressions of the senescence-related proteins p53 and p-p53 in BMMSCs cultured in vitro from aged mice (**e**) and young mice (**f**) were examined by western blotting. The data are presented as the means ± SD of each independent experiment performed in triplicate. *P < 0.05, **P < 0.01, ***P < 0.001, one-way analysis of variance (ANOVA)

To assess the effect of HDAC9 on aged BMMSC differentiation, HDAC activity was inhibited in BMMSCs using the histone deacetylase inhibitors Trichostatin A (TSA), sodium butyrate (NaB), and *HDAC9* siRNA (siHDAC9) during osteogenic and adipogenic differentiation. Firstly, BMMSCs were treated with different doses of TSA (50, 100, and 200 nmol/L) and NaB (50, 100, 200, and 400 μmol/L) to examine changes in H3K9 acetylation. Western blotting results showed that 100 nmol/L and 200 nmol/L of TSA (sFig. 3a), and 200 μmol/L and 400 μmol/L of NaB (sFig. 3b) effectively enhanced H3K9 acetylation level in BMMSCs. However, much apoptosis occurs in cells administrated with 200 nmol/L TSA and 400 μmol/L NaB, respectively. Hence, 100 nmol/L of TSA and 200 μmol/L of NaB were chosen as the working concentration. The results of Alizarin Red staining and western blotting analysis of osteogenic associated proteins showed that treatment with TSA or NaB significantly restored the osteogenic differentiation of aged BMMSCs (sFig. 3c). Oil Red O staining and western blotting analysis of adipogenic associated protein PPAR-γ displayed that TSA or NaB effectively inhibited the adipogenic differentiation of aged BMMSCs (sFig. 3d). Next, we used *HDAC9* siRNA to specially inhibit HDAC9 expression in aged BMMSCs (sFig. 4). We found that HDAC9 inhibition promoted osteogenic differentiation and repressed adipogenic differentiation in aged BMMSCs (Fig. 2a, b) and reduced the expression of senescence-related proteins p53 and p-p53 (Fig. 2e). To explore whether the effect of HDAC9 on BMMSC differentiation is age dependent, *HDAC9* was silenced by siRNA in young BMMSCs. These results showed that HDAC9 regulated lineage differentiation in young BMMSCs (Fig. 2c, d) and the expression of senescence-related proteins (Fig. 2f), but not as effective as in aged BMMSCs. Above all, these results indicated that inhibition of HDCA9 converted BMMSCs into a younger state, with partially restored lineage differentiation balance and reduced expression of senescence-related proteins. However, the underlying mechanism of how HDAC9 regulates the differentiation of aged BMMSCs remained unclear.

### Autophagy decreased in BMMSCs derived from aged mice

**Fig. 3:**
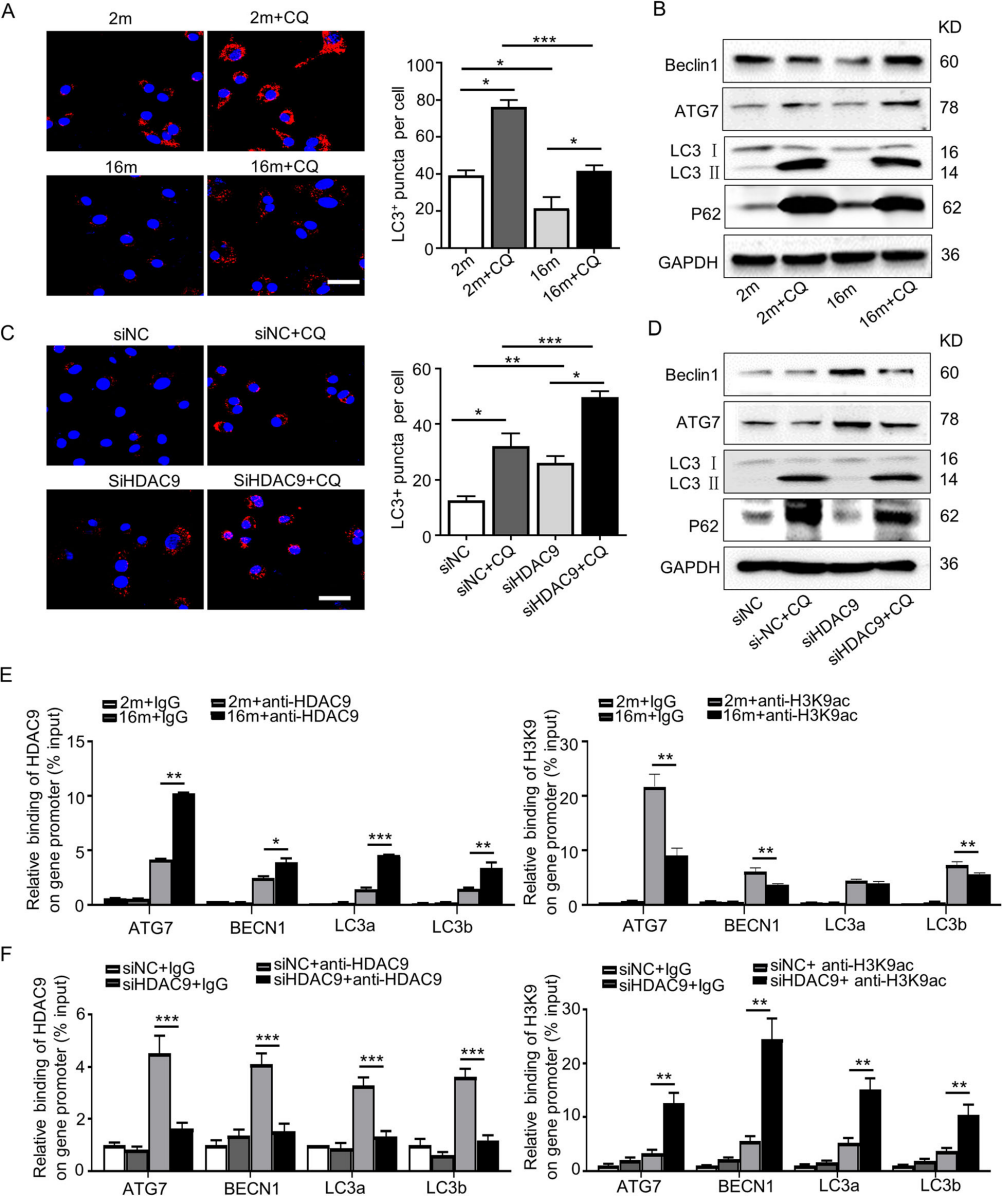
HDAC9 regulated the autophagy in BMMSCs by directly binding to the promoters of autophagy-related genes. To evaluate the role of HDAC9 in regulating autophagy, young and aged BMMSCs cultured in vitro and aged BMMSCs were transfected with Nc siRNA or *HDAC9* siRNA. **a** LC3 was measured by immunofluorescence staining in young and aged BMMSCs, and those cells treated with CQ. Scale bars = 50 μm. **b** Autophagy-related proteins were detected in young and aged BMMSCs, and those cells treated with CQ by western blotting. **c** LC3 was measured by immunofluorescence staining in aged BMMSCs transfected with Nc siRNA or *HDAC9* siRNA, and those cells treated with CQ. Scale bar = 50 μm. **d** Autophagy-related proteins were detected in aged BMMSCs transfected with Nc siRNA or *HDAC9* siRNA, and those cells treated with CQ by western blotting. **e**, **f** The chromatin immunoprecipitation (ChIP) assay was performed to investigate whether HDAC9 could bind with the promoters of autophagy-related genes. Chromatin was isolated from young and aged BMMSCs (**e**) and aged BMMSCs transfected with Nc siRNA or *HDAC9* siRNA (**f**), and incubated with HDAC9, acetylated-histone H3K9 (H3K9ac), and IgG antibodies. An IgG antibody was used as a negative control. Immunoprecipitation with specific HDAC9 or H3K9ac antibody. Data are presented as the mean ± SD of triplicate samples. *P < 0.05, **P < 0.01, ***P < 0.001. **a**, **c** One-way analysis of variance (ANOVA). **e**, **f** Unpaired two-tailed Student’s *t* test

Our previous study showed that autophagy is a key regulator of BMMSC differentiation in OVX-induced osteoporosis and aged-induced bone mass loss [20, 21]. To evaluate changes in autophagy activity in BMMSCs, the autophagy-flux inhibitor, chloroquine (CQ), was used to prevent autophagy-lysosome degradation during active autophagy [22]. The transmission electron microscope (TEM) results showed that aged BMMSCs possessed fewer autophagosomes than young cells, especially when treated with CQ (sFig. 5a). Furthermore, immunofluorescence staining results showed that the number of LC3 (an autophagy marker)-positive cells significantly decreased in aged BMMSCs (Fig. 3a). Meanwhile, the expression levels of the autophagy-related proteins, ATG7, Beclin1, and LC3II/I, were reduced in aged BMMSCs (Fig. 3b). However, the expression level of p62, the substrate of selective autophagy, increased in aged BMMSCs (Fig. 3b). These data suggested impaired autophagic activity in aged cells.

### HDAC9 impaired the differentiation of aged BMMSCs by regulating autophagy

As the role of histone deacetylases involved in autophagy has previously been explored [23, 24], we next investigated the role of HDAC9 in the epigenetic regulation of autophagy in BMMSCs. To confirm whether HDAC9 could regulate autophagy in aged BMMSCs, we silenced *HDAC9* expression using *HDAC9* siRNA. The results showed HDAC9 expression significantly decreased and H3K9 acetylation levels increased in aged BMMSCs transfected with *HDAC9* siRNA (sFig. 6). Next, we found that *HDAC9* siRNA treatment increased the number of LC3-positive cells (Fig. 3c) and autophagosomes (sFig. 5b) in aged BMMSCs. These effects of *HDAC9* siRNA were amplified when aged cells were treated with CQ. In addition, the protein expression levels of ATG7, Beclin1, and LC3II/I were higher and protein expression levels of p62 were lower in BMMSCs treated with *HDAC9* siRNA or CQ, compared with that in control cells (Fig. 3d). These results indicated that HDAC9 has a close relationship with autophagy.

To test the hypothesis that HDAC9 regulates autophagy by modulating the acetylation of H3K9 associated with autophagy-related genes, chromatin immunoprecipitation (ChIP) assays were performed in BMMSCs. ChIP analysis was performed using antibodies that individually recognize either acetylated H3K9 (H3K9ac) or HDAC9, and the primers were used to amplify the promoter regions of *Atg7*, *BECN1*, *LC3a*, and *LC3b* (Supplementary Table 2). First, the effect of HDAC9 binding to autophagy-related genes was examined in young and aged BMMSCs. ChIP results showed that the level of HDAC9 binding to the promoters of *Atg7*, *BECN1*, *LC3a*, and *LC3b* significantly increased in aged BMMSCs. In contrast, the level of H3K9ac binding to these promoters was significantly reduced in aged BMMSCs (Fig. 3e). To confirm that decreased H3K9 acetylation in the promoters of *Atg7*, *BECN1*, *LC3a*, and *LC3b* genes was dependent on HDAC9 expression, *HDAC9* was knocked down by *HDAC9* siRNA in aged BMMSCs. We found that HDAC9 binding to the promoters of these genes was blocked in aged BMMSCs transfected with an *HDAC9* siRNA, and subsequently, the level of H3K9 acetylation at these promoters increased (Fig. 3f). Overall, these results indicated that HDAC9 regulates autophagy in BMMSCs by controlling the acetylation of H3K9 associated with autophagy-related genes.

**Fig. 4:**
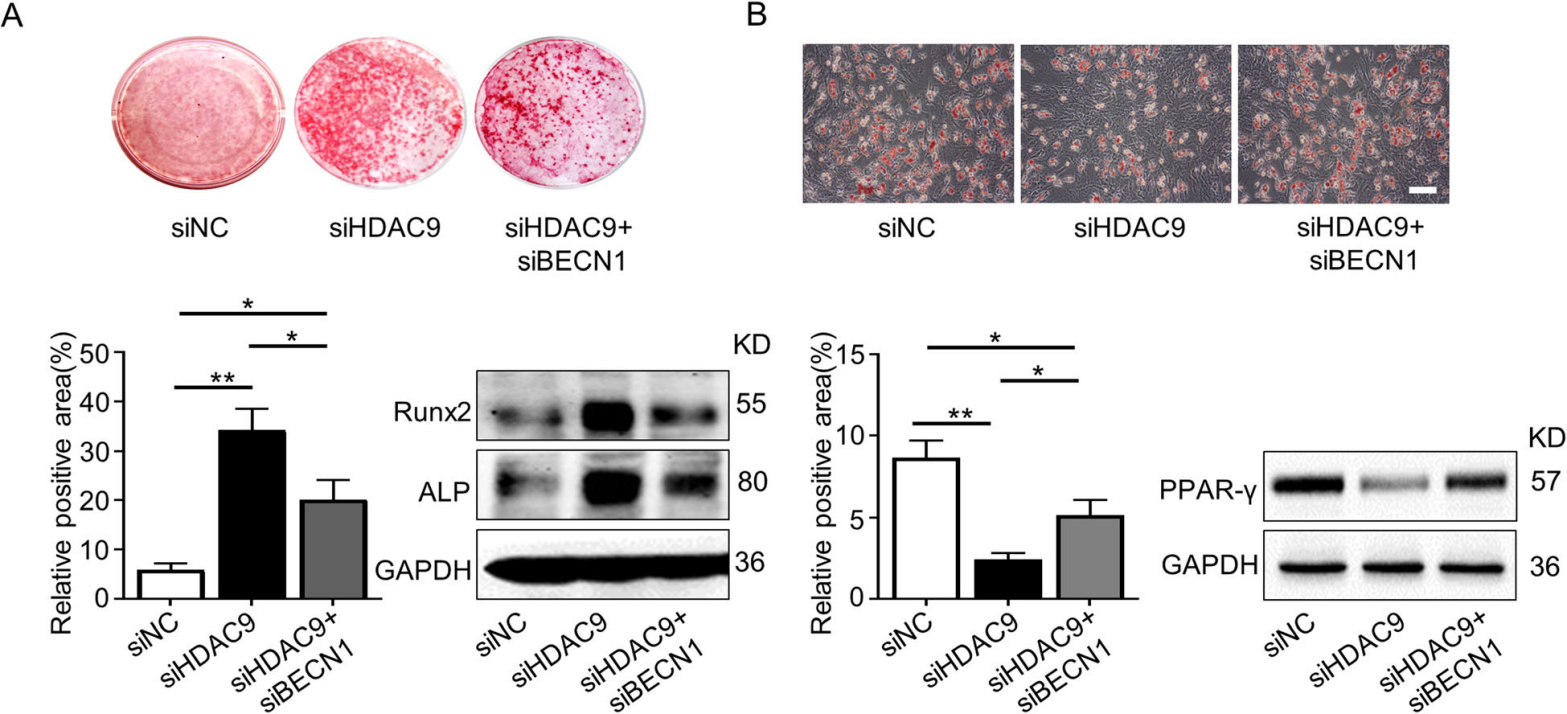
Inhibition of autophagy blocked the ability of HDAC9 siRNA to rebalance BMMSC differentiation. To investigate the HDAC9-autophagy axis regulating BMMSC function, BMMSCs were respectively transfected with Nc siRNA and *HDAC9* siRNA or co-transfected with *HDAC9* siRNA and *BECN1* siRNA. **a** Alizarin Red staining was performed, and osteogenesis-related proteins were analyzed in aged BMMSCs from above three groups. **b** Oil Red O staining was performed, and adipogenesis-related proteins were analyzed in three groups of cells described above. Scale bars = 100 μm. The data are presented as the means ± SD of each independent experiment performed in triplicate. *P < 0.05, **P < 0.01, one-way analysis of variance (ANOVA)

Given that *HDAC9* siRNA effectively improved aged BMMSC differentiation and that HDAC9 regulates autophagy, we next examined whether HDAC9 affect the differentiation of aged BMMSCs by regulating autophagy. We silenced *HDAC9* and *BECN1*, a key gene in the regulation of autophagosome formation [25, 26], in aged BMMSCs (sFig. 7). The effect of *HDAC9* siRNA on improving the osteogenic differentiation of aged BMMSCs was neutralized by silencing *BECN1* expression (Fig. 4a). Conversely, Oil Red O staining and western blotting results showed that the adipogenic differentiation of aged BMMSCs increased when cells were co-transfected with both *HDAC9* siRNA and *BECN1* siRNA, compared with cells transfected with *HDAC9* siRNA only (Fig. 4b). Collectively, these results showed that the HDAC9-induced imbalance in the differentiation of aged BMMSCs was partially attributed to impaired autophagy.

### Inhibition of HDAC9 improved bone mass loss in aged mice

**Fig. 5:**
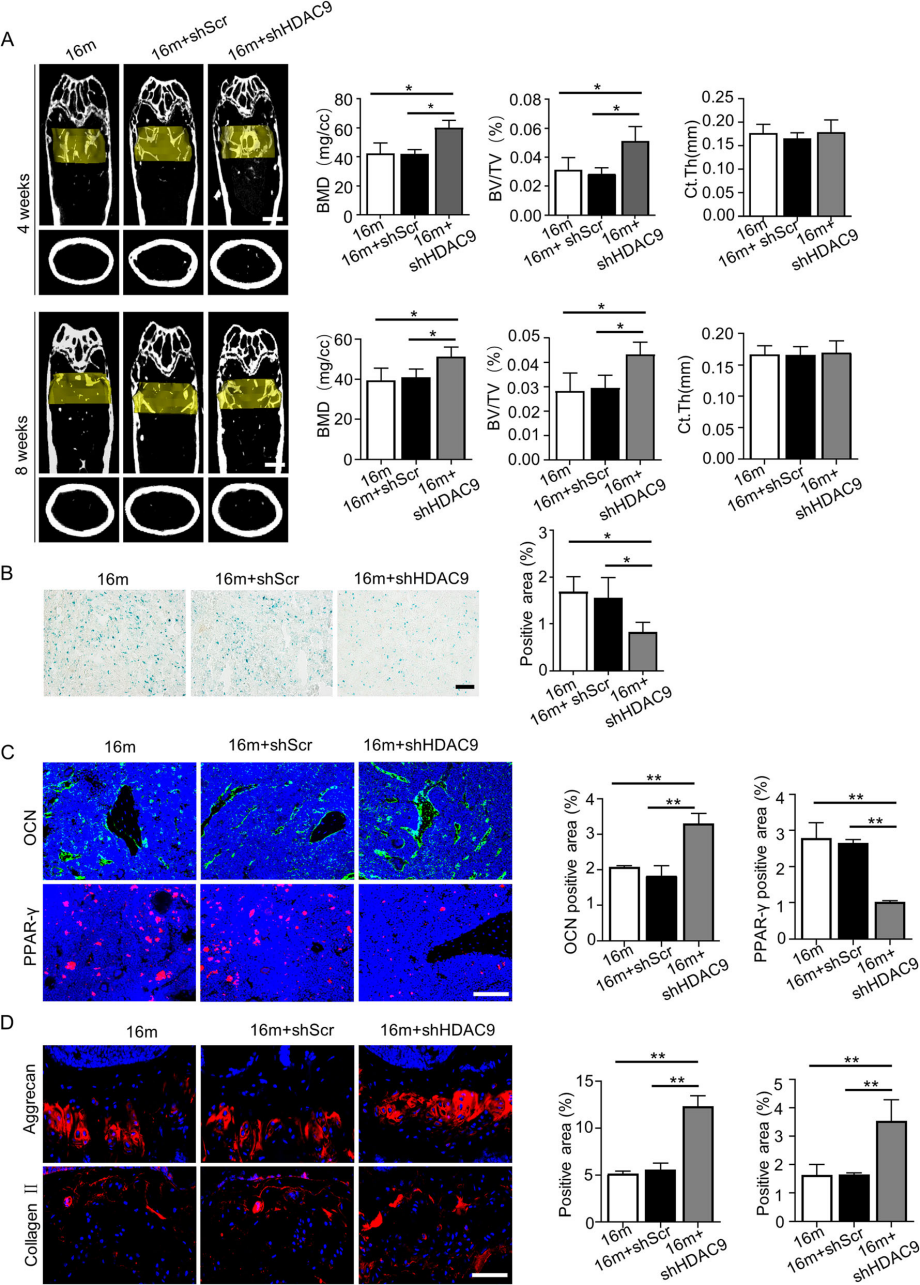
Inhibition of HDAC9 partially restores bone loss in aged mice. **a** Micro-CT analysis of trabecular bone mass and cortical bone thickness in the femora of aged mice from the control, shScr-treated, and shHDAC9-treated groups was examined 4 weeks and 8 weeks operation, respectively. Quantitative analysis of bone mineral density (BMD), trabecular bone volume (BV/TV), and cortical bone thickness (Ct.Th) was performed. Scale bar = 1 mm. **b** SAbetaGal staining was performed in the bone marrow of mice from the control, shScr-treated, and shHDAC9-treated groups 4 weeks after treatment, and SAbetaGal positive signals were quantitatively analyzed. Scale bar = 100 μm. **c** Immunofluorescent staining of OCN and PPAR-γ was performed in the femora 4 weeks after treatment, and quantitative relative positive ratio areas of the femora were analyzed. Scale bars = 100 μm. **d** Immunofluorescent staining of aggrecan and collagen II was performed in the femora 4 weeks after administration, and quantitative relative positive ratio areas of the femora were analyzed. Scale bars = 50 μm. *n* = 7 mice per group. The data are analyzed with one-way analysis of variance (ANOVA) and presented as the means ± SD of each independent experiment performed in triplicate. *P < 0.05, **P < 0.01, ***P < 0.001

To determine whether HDAC9 functions in vivo, we used a lentivirus containing *HDAC9* shRNA to inhibit HDAC9 expression in the bone marrow of aged mice with senile osteoporosis. Micro-CT analysis was performed to examine the bone mass of control mice, shHDAC9-treated mice, and shScr-treated mice. The results showed that improved bone mass (increased BMD, BV/TV) of aged mice was observed 4 weeks or 8 weeks after *shHDAC9* treatment (Fig. 5a). Moreover, no further improvement of bone mass was detected 8 weeks compared with 4 weeks. The bone mass of all groups underwent slight decrease in the further 1 month. SAbetaGal and immunofluorescence staining of PPAR-γ showed that the number of senescence cells and adipocytes reduced and the number of OCN-positive cells increased in the bone marrow of aged mice after *HDAC9* inhibition (Fig. 5b, c). Considering chondrocytes are important in bone formation, we detected the chondrocytes in growth plate after inhibition of HDAC9. The Alcian blue staining showed that inhibition of HDAC9 had no significant improvement on the thickness of growth plate (sFig. 8), but promoted the expression of aggrecan and collagen II in chondrocytes (Fig. 5d).

**Fig. 6:**
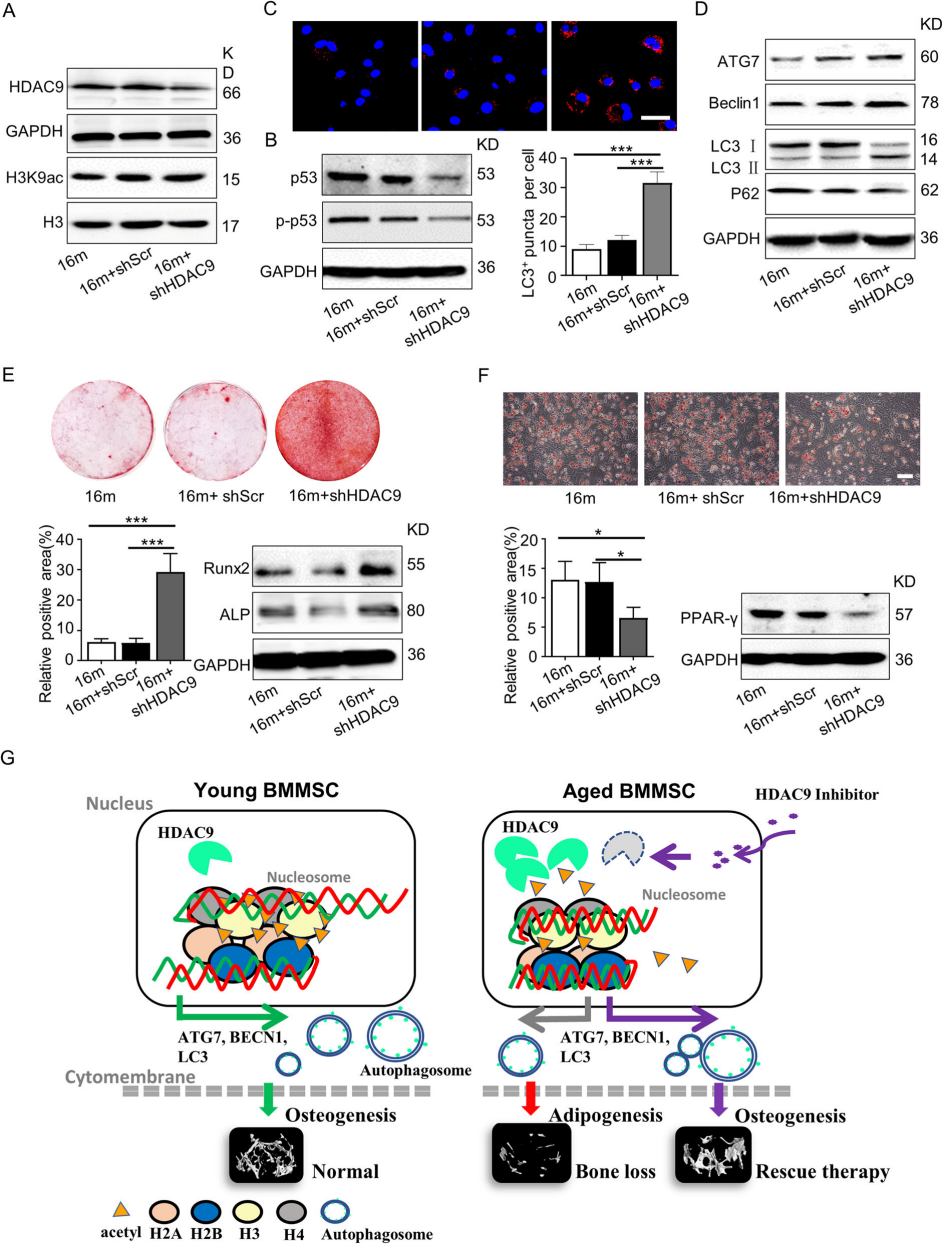
Inhibition of HDAC9 improved the lineage differentiation of endogenous BMMSCs ex vivo. The BMMSCs were harvested from the mice from the control, shScr-treated, and shHDAC9-treated groups 4 weeks after bone intrainjection. **a** Western blotting was performed to analyze the expressions of HDAC9 and the acetylation of H3K9 in BMMSCs from the three groups. **b** Expression of senescence-related proteins p53 and p-p53 in BMMSCs by western blotting. **c** LC3 was measured by immunofluorescence staining from all groups of aged BMMSCs. Scale bars = 50 μm. **d** The expression of autophagy-related proteins in BMMSCs were examined by western blotting. **e** Alizarin Red staining was performed, and osteogenesis-related proteins were detected in BMMSCs from the three groups. **f** Oil Red O staining was performed, and adipogenesis-related protein were detected in BMMSCs from the control, shScr-treated, and shHDAC9-treated groups. Scale bars = 100 μm. **g** Schematic diagram depicts how HDAC9 regulates BMMSC differentiation via controlling autophagy and a therapeutic method. In young BMMSCs, the low expression level of HDAC9 maintains the high levels of acetylation modifications on H3K9 of autophagy-related genes which promotes intracellular autophagosomes formation, and subsequently facilitates osteogenic differentiation of BMMSCs. In aged BMMSCs, increased HDAC9 expression leads to deacetylation of H3K9 of autophagy-related genes, which inhibits intracellular autophagosome formation. Insufficient autophagy subsequently promotes adipogenic differentiation, inhibits osteogenic differentiation of BMMSCs, and ultimately leads to bone mass loss. shHDAC9 treatment could partially rescue the impaired osteogenic differentiation of aged endogenous BMMSCs and restore bone mass. The data are presented as the means ± SD of each independent experiment performed in triplicate. *P < 0.05, ***P < 0.001, one-way analysis of variance (ANOVA)

Then, primary BMMSCs from control, shHDAC9-treated, and shScr-treated mice were cultured and detected. Western blotting was used to detect H3K9 acetylation and the expression of senescence- and autophagic-associated proteins in BMMSCs. BMMSCs from shHDAC9-treated aged mice were shown to have increased H3K9 acetylation (Fig. 6a), decreased protein expression levels of p53 and p-p53 (Fig. 6b), and increased the number of LC3-positive cells and the expressions levels of ATG7 and Beclin1 (Fig. 6c, d). In addition, the osteogenic and adipogenic differentiation capacities of BMMSCs were evaluated. Alizarin Red staining and western blotting results showed that osteogenesis of BMMSCs significantly improved from shHDAC9-treated mice (Fig. 6e). Furthermore, Oil Red O staining and the expression of adipogenesis-associated protein PPAR-γ demonstrated shHDAC9-treated BMMSCs had a lower level of adipogenic differentiation than control cells (Fig. 6f). In summary, these data suggested that the inhibition of HDAC9 could improve BMMSC lineage differentiation imbalance and ameliorate bone mass loss in aged mice. Our results indicated that the inhibition of HDAC9 could alleviate aged-related bone mass loss in mice, which suggests its potential as a target in the clinical treatment of senile osteoporosis (Fig. 6g).

## Discussion

The effects of HDACs on bone formation, bone absorption, and regeneration have been studied over the last few decades. The conditional deletion of *HDAC3* in osteochondral progenitors using Osx1-Cre or in osteoblasts using OCN-Cre leads to cortical and trabecular bone mass loss and increased marrow adiposity [27, 28]. Some studies have demonstrated reduced bone mass and increased bone resorption in *HDAC4*- [19], *HDAC5*- [29], *HDAC7*- [17], and *HDAC9*-deficient [18] mice. Conversely, *HDAC6*-deficient mice showed increased bone mineral density [30]. Although various *HDAC*-deficient animal models have been established to investigate bone remodeling, the effects on aged-related bone mass loss and the associated mechanisms remain unclear.

In this study, of the HDAC family members detected, only HDAC9 increased and acetylation of H3K9 consequently decreased in the bone and muscle of aged mice compared with young mice. The same results were also observed in senescent BMMSCs. Our previous works found that HDAC9 expression significantly increased in human periodontal ligament stem cells (PDLSCs) under inflammatory microenvironment [31]. Kofman et al. showed that HDAC9 expression increased in aging spermatogonial stem cells and then decreased when cells were exposed to the anti-aging drug, rapamycin [32], which is consistent with our findings. HDAC inhibitors have been proposed as promising anti-aging drugs. In this study, we found modulating HDAC9 expression in BMMSCs could regulate the levels of the senescence-related proteins p53 and p-p53. In addition, increased HDAC9 expression impaired the osteogenesis capacity of both aged BMMSCs and inflammatory PDLSCs [31]. Meanwhile, increased HDAC9 expression facilitated adipogenic differentiation in BMMSCs. Inhibition of *HDAC9* by siRNA transfection improved the lineage differentiation capacity and decreased the senescence of aged BMMSCs, whereas *HDAC9* inhibition in young BMMSCs did not display as effective as in aged BMMSCs, possibly because the level of HDAC9 expression was relatively low in young BMMSCs. Interestingly, some studies reported that HDAC9 regulates the osteogenic differentiation of MSCs [33, 34]. Chen et al. showed that HDAC9 expression decreased in old mice and this was associated with bone aging [34], which contradicts our observations in aged mice. In our study, HDAC9 expression was compared in the bone marrow of 2-month-old and 16-moonth-old female mice. However, Chen et al. compared HDAC9 expression in MSCs from mice in their study which were designed less than 7-month-old as young mice and more than 7-month-old as old mice, with no details information of the sex. There are known to be sex differences in the pathogenesis and mechanisms of senile osteoporosis. Furthermore, the MSCs were performed for functional verification of HDAC9 which was different. Chen and colleagues used MSCs derived from human; however, the MSCs used were derived from mice in this study. These differences in observation time points and cell genus may be key factors leading to contrasting results.

Autophagy is a very important degradation system, playing a crucial role in maintaining cell homeostasis in response to cellular stress [35, 36]. Autophagy dysfunction is associated with several diseases, such as inflammation, cancer, neurodegeneration, and aging [36]. Recent studies have indicated that autophagy is a key process in bone cell differentiation. It has been reported that autophagy can regulate osteoclast and chondrocyte differentiation [37] and protect BMMSCs from oxidative stress [38], which indicates that autophagy plays protective roles in maintaining bone homeostasis. Our previous study also showed that impaired autophagy triggers intracellular ROS-induced senescence in BMMSCs, which shifts cellular lineage commitment from osteoblasts to adipocytes, consequently leading to bone aging [20, 21]. This is consistent with the mainstream views that autophagy acts as a crucial stem cell fate regulator by controlling ROS associated with p16 and p21 [22, 39]. However, the upstream factors regulating autophagy in BMMSCs are still unclear and need to be further investigated.

Epigenetic modifications associated with the aging process have received our attention over the past few decades. Epigenetic regulations have been widely studied in somatic stem cells to help us understand its roles in governing self-renewal or lineage differentiation. Meanwhile, some studies have reported epigenetic modifications, including acetylation of H4K16 associated with ATG genes [24, 40], regulation of *LC3* transcription by histone methyl-transferase (HMT) G9a [23, 41], and hypermethylation of the *BECN1* promoter [42], which is involved in regulating the formation of autophagosomes. To date, very few studies have detected histone deacetylases in MSCs to explore whether changes in epigenetic modification are associated with autophagy during bone aging. Here, we demonstrated that increased HDAC9 expression impaired autophagy activity in aged BMMSCs. Furthermore, we found that HDAC9 directly interact with the promoter of autophagy-related genes, *ATG7*, *BECN1*, and *LC3a/b*, by ChIP analysis. Inhibition of HDAC9 expression in aged BMMSCs by siRNA transfection reduced HDAC9 binding to the promoters of *Atg7*, *BECN1*, *LC3a*, and *LC3b*, but increased the levels of acetylated H3H9 interaction in the promoter of autophagy-related genes. Thus, we speculated the increased expression of HDAC9 in aged BMMSCs is an important factor leading to autophagic degeneration and this finding may uncover a new mechanism for the regulation of aged-related bone mass loss by HDAC9. However, there are still some unanswered questions. For example, the animal model we used was aged female mice with postmenopausal osteoporosis, which may not have an identical pathogenesis to aged male mice. Therefore, further investigation is required to determine whether the mechanism identified in this study is also applicable to aged male mice. Furthermore, HDAC9 was found to affect bone resorption [18], which is a key factor in maintaining the balance of bone remodeling. We need to explore the HDAC9-mediated osteoclastogenesis in bone remodeling in more detail in future experiments and identify the regulatory networks operating between BMMSCs and osteoclasts.

## Conclusions

Taken together, our study suggested a potential role and mechanism of HDAC9 in BMMSC lineage commitment by regulating autophagy activities. Knockdown *HDAC9* improved endogenous BMMSC properties and promoted the bone mass recovery in aged mice. Our finding provided a potentially promising target for the prevention of osteoporosis and treatment of aged-related bone mass loss.

## Supplementary information

**Additional file 1: sFig. 1** The expressions of HDACs family members in bone marrow and BMMSCs HDACs gene were examined in bone marrow (a) and BMMSCs (b) from young mice and aged mice by qRT-PCR. **sFig. 2** The expressions of senescence associated proteins and HDAC9 in skeletal muscle and myoblasts. The expression of p53, HDAC9 and H3K9 acetylation, and *HDAC9* in skeletal muscle (a-c) and myoblasts (d-f) were examined by western blotting and qRT-PCR from young mice and aged mice. **sFig. 3** HDACs inhibitors promoted osteogenic differentiation and inhibited adipogenic differentiation in aged BMMSCs The expression of HDAC9 and H3K9 acetylation were detected in aged BMMSCs treatment with different dose of TSA (a) or NaB (b) by western blotting. Osteogenic differentiation (c) and adipogenic differentiation (d) were observed in aged BMMSCs treated with TSA or NaB. Scale bars = 100 μm. **sFig. 4** The silence efficiency of *HDAC9* siRNA HDAC9 and H3K9 acetylation were examined in aged BMMSCs after transfected with *HDAC9* siRNA for 48 hours, 3 days and 7days by qRT-PCR (a, d) and western blotting (b, c, e). **sFig. 5***HDAC9* siRNA restored the number of autophagosomes in aged BMMSCs Autophagosomes in young, aged BMMSCs (a) and aged BMMSCs transfected with *HDAC9* siRNA (b) were detected by TEM. Scale bars = 1 μm. **sFig. 6***HDAC9* siRNA decreased H3K9 acetylation in aged BMMSCs. The expression of HDAC9 and H3K9 acetylation were examined by western blotting in aged BMMSCs transfected with *HDAC9* siRNA and those cells treated with CQ. **sFig. 7** The silence efficiency of BECN1 *siRNA* Beclin1 was examined by western blotting in aged BMMSCs transfected with *BECN1* siRNA 48 hours later. **sFig. 8** No changes in growth plate thickness in aged mice were treated with *HDAC9* shRNA lentivirus Alcian blue staining were performed to assess growth plate thickness.

**Additional file 2: Supplementary Table 1**. Primer sequences for Real-Time PCR assay. **Supplementary Table 2**. Primer sequences for ChIP assay.

## Data Availability

All datasets used and/or analyzed during the current study are available from the corresponding author on reasonable request.

## Abbreviation

BMMSCs: Bone marrow mesenchymal stem cells
HDACs: Histone deacetylases
ROS: Reactive oxygen species
DNMTs: DNA methyltransferases
Runx2: Runt-related transcription factor 2
ALP: Alkaline phosphatase
PPARγ: Peroxisome proliferator activated receptor gamma
OCN: Osteocalcin
TRAP: Tartrate-resistant acid phosphatase
ATG7: Autophagy-related 7
BECN1: Beclin 1
LC3: Microtubule associated protein 1 light chain 3
p62: Ubiquitin-binding protein p62
H3: Histone H3
H3K9: Histone H3 lysine 9
H3K9ac: Acetyl-histone H3 lysine 9
H3K14: Histone H3 lysine 14
H3K18: Histone H3 lysine 18
p53: Tumor protein p53
TSA: Trichostatin A
NaB: Sodium butyrate
CQ: Chloroquine
micro-CT: Micro-computed tomography
qRT-PCR: Real-time polymerase chain reaction
ChIP: Chromatin immunoprecipitation
SAbetaGal: Senescence-associated β-galactosidase
