# Correction: Impaired autophagy triggered by HDAC9 in mesenchymal stem cells accelerates bone mass loss

**DOI:** 10.1186/s13287-022-03080-y

**Published:** 2022-07-27

**Authors:** Liqiang Zhang, Meng Qi, Ji Chen, Jiangdong Zhao, Liya Li, Jiachen Hu, Yan Jin, Wenjia Liu

**Affiliations:** 1 National & Local Joint Engineering Research Center of Biodiagnosis and Biotherapy, Precision Medicine Institute, The Second Affiliated Hospital of Xi’an Jiaotong University, Xi’an, 710004 China; 2 State Key Laboratory of Military Stomatology & National Clinical Research Center for Oral Diseases & Shaanxi International Joint Research Center for Oral Diseases, Center for Tissue Engineering, School of Stomatology, The Fourth Military Medical University, No. 145 West Changle Road, Xi’an, 710032 Shaanxi China; 3 Xi’an Institute of Tissue Engineering and Regenerative Medicine, Xi’an, 710032 Shaanxi China; 4 The Key Laboratory of Aerospace Medicine, Ministry of Education, The Fourth Military Medical University, Xi’an, 710032 Shaanxi China

## Correction to: Stem Cell Research & Therapy (2020) 11:269 10.1186/s13287-020-01785-6

Following the publication of the original article [1], the authors identified image overlap in Figs. 2d, 3c and 6c. The image misuse was due to mislabeling of the original data. The authors checked the original data and wish to replace the representative Oil red O staining image of siHDAC9 in Fig. 2d and the LC3 immunofluorescence staining of 16 m + shSCr in Fig. 6c.

**Fig. 2:**
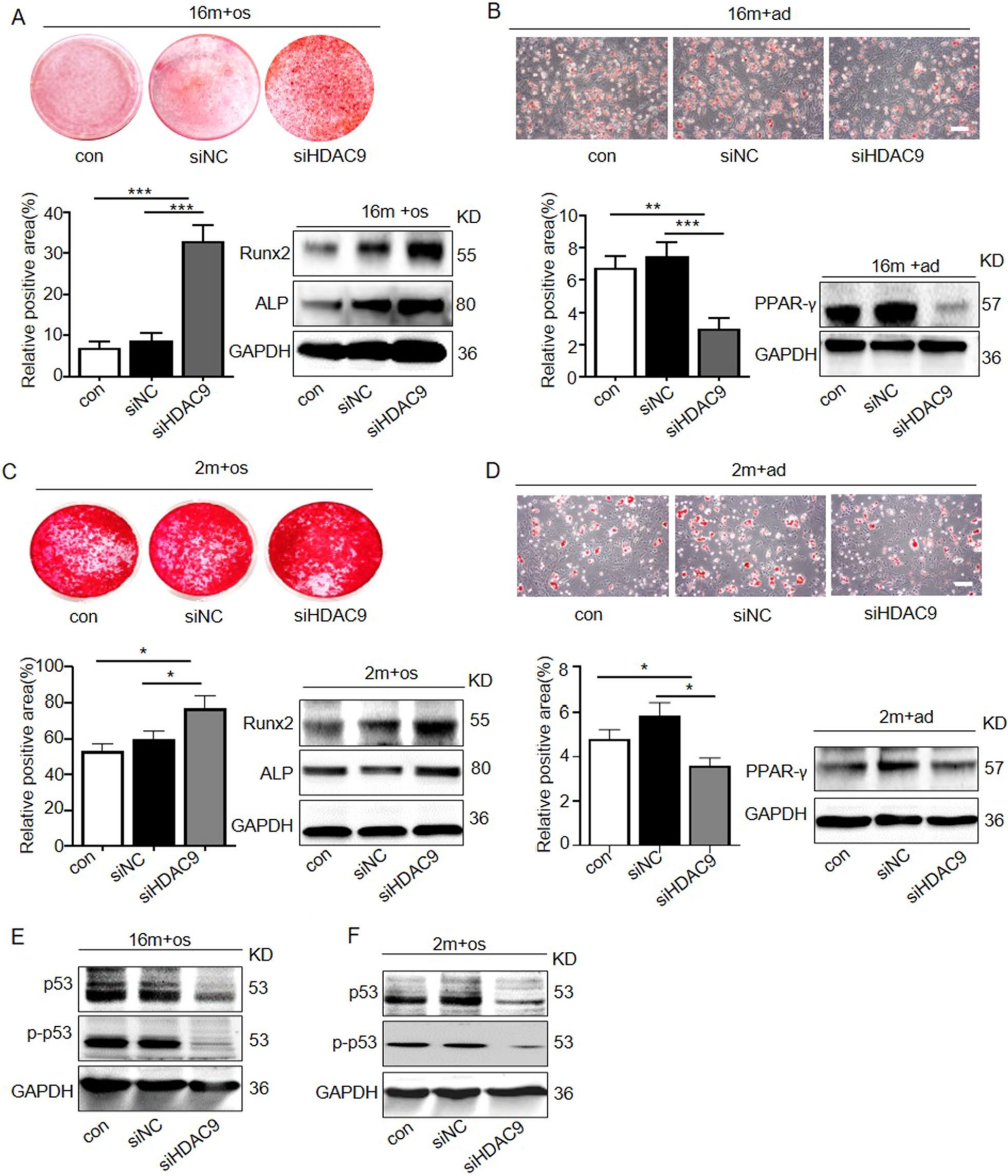
Downregulation of HDAC9 rescued lineage differentiation imbalance and ameliorated senescence in aged BMMSCs. **a** Alizarin Red staining was performed, and osteogenesis-related proteins were detected by western blotting in aged BMMSCs transfected with HDAC9 siRNA. **b** Oil Red O staining was performed, and adipogenesis-related proteins were detected by western blotting in aged BMMSCs transfected with HDAC9 siRNA. **c** Alizarin Red staining was performed, and osteogenic-related proteins were detected by western blotting in young BMMSCs and young BMMSCs transfected with HDAC9 siRNA. **d** Oil Red O staining was performed, and adipogenic-related proteins were detected by western blotting in young BMMSCs and young BMMSCs transfected with HDAC9 siRNA. **e**, **f** Expressions of the senescence-related proteins p53 and p-p53 in BMMSCs cultured in vitro from aged mice (**e**) and young mice (**f**) were examined by western blotting. The data are presented as the means ± SD of each independent experiment performed in triplicate. *P < 0.05, **P < 0.01, ***P < 0.001, one-way analysis of variance (ANOVA)

**Fig. 6:**
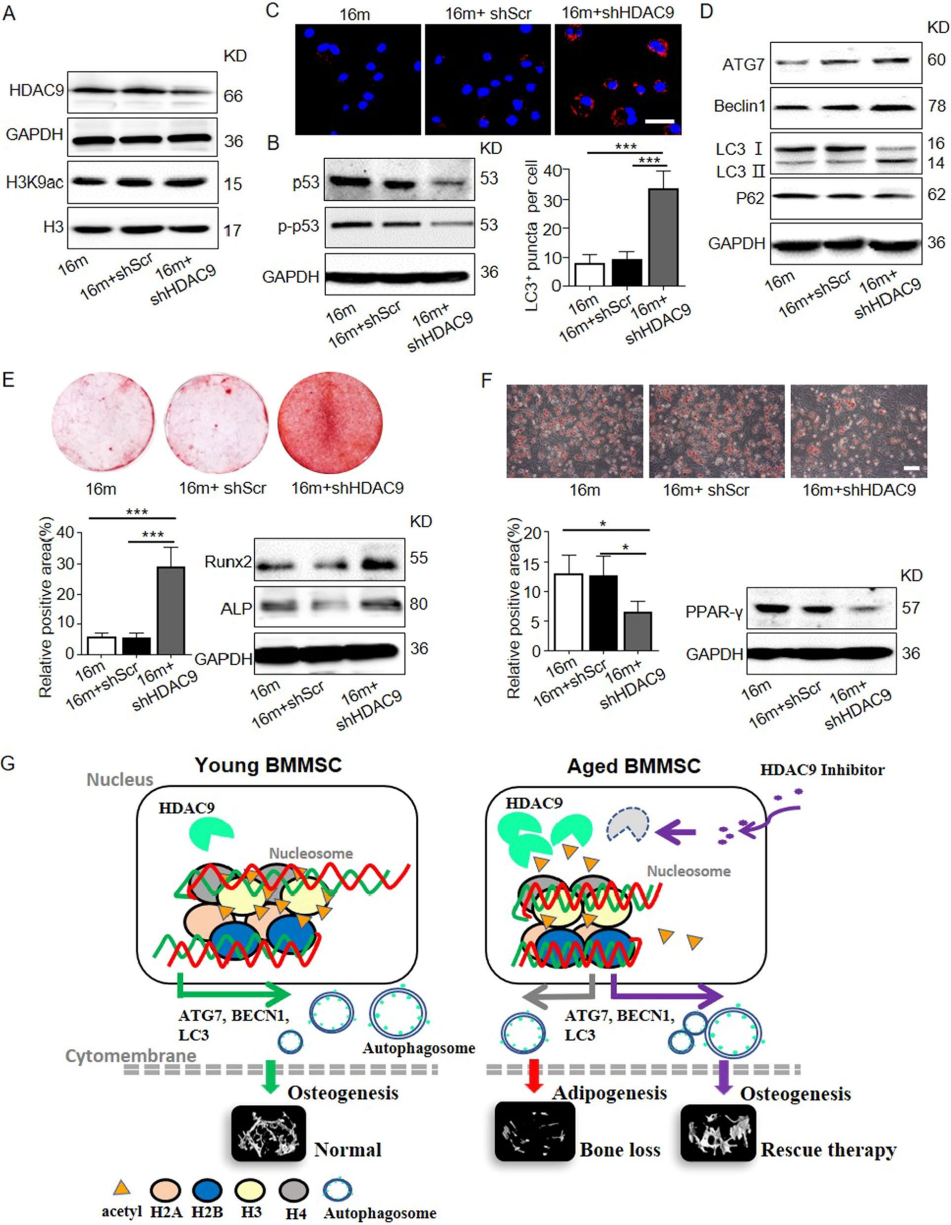
Inhibition of HDAC9 improved the lineage differentiation of endogenous BMMSCs ex vivo. The BMMSCs were harvested from the mice from the control, shScr-treated, and shHDAC9-treated groups 4 weeks after bone intrainjection. **a** Western blotting was performed to analyze the expressions of HDAC9 and the acetylation of H3K9 in BMMSCs from the three groups. **b** Expression of senescence-related proteins p53 and p-p53 in BMMSCs by western blotting. **c** LC3 was measured by immunofluorescence staining from all groups of aged BMMSCs. Scale bars = 50 μm. **d** The expression of autophagy-related proteins in BMMSCs were examined by western blotting. **e** Alizarin Red staining was performed, and osteogenesis-related proteins were detected in BMMSCs from the three groups. **f** Oil Red O staining was performed, and adipogenesis-related protein were detected in BMMSCs from the control, shScr-treated, and shHDAC9-treated groups. Scale bars = 100 μm. **g** Schematic diagram depicts how HDAC9 regulates BMMSC differentiation via controlling autophagy and a therapeutic method. In young BMMSCs, the low expression level of HDAC9 maintains the high levels of acetylation modifications on H3K9 of autophagy-related genes which promotes intracellular autophagosomes formation, and subsequently facilitates osteogenic differentiation of BMMSCs. In aged BMMSCs, increased HDAC9 expression leads to deacetylation of H3K9 of autophagy-related genes, which inhibits intracellular autophagosome formation. Insufficient autophagy subsequently promotes adipogenic differentiation, inhibits osteogenic differentiation of BMMSCs, and ultimately leads to bone mass loss. shHDAC9 treatment could partially rescue the impaired osteogenic differentiation of aged endogenous BMMSCs and restore bone mass. The data are presented as the means ± SD of each independent experiment performed in triplicate. *P < 0.05, ***P < 0.001, one-way analysis of variance (ANOVA)

These corrections do not affect the original conclusions. The authors apologize for any inconvenience that the errors may have caused.

The correct figures have been included in this correction.
