# Supplementary material for: Impaired autophagy triggered by HDAC9 in mesenchymal stem cells accelerates bone mass loss
Source: Stem Cell Res Ther. 2020 Jul 3;11:269. doi: 10.1186/s13287-020-01785-6 (PMC7333327; doi:10.1186/s13287-020-01785-6)

Supplementary Figure legends

sFig. 1 The expressions of HDACs family members in bone marrow and BMMSCs from young and aged mice

a, b Expressions of HDACs were examined in bone marrow (a) and BMMSCs (b) from young mice compared with those from aged mice by qRT-PCR. *P < 0.05, **P < 0.01, ***P < 0.001, unpaired two-tailed Student's t-test.

sFig. 2 The expressions of senescence associated proteins and HDAC9 in skeletal muscle and myoblasts

a, d p53, p-p53, in skeletal muscle (a) and myoblasts (d) from young and aged mice were examined by western blotting. b, e The expression of *HDAC9* in skeletal muscle (b) and myoblasts (e) from young and aged mice were examined by RT-PCR. c, f The expressions of HDAC9 and the levels of acetylation of H3K9 in skeletal muscle (c) and myoblasts (f) from young and aged mice were examined by western blotting. Data are the mean ± s.d. of triplicate samples. *P < 0.05, **P < 0.01, a, c unpaired two-tailed Student's t-test.

sFig. 3 HDACs inhibitors treatment promoted osteogenic differentiation and inhibited adipogenic differentiation in BMMSCs

a, b BMMSCs were treated with different dose of HDAC9 inhibitors (TSA (a) or NaB (b)), and the expression of HDAC9 and the levels of acetylation of H3K9 were detected by western blotting. c Alizarin Red staining was performed and osteogenesis-related proteins were detected with western blotting in aged BMMSCs treated with HDACs inhibitors, TSA or NaB. d Oil Red O staining was performed and adipogenesis-related proteins were detected with western blotting in aged BMMSCs treated with HDACs inhibitors, TSA or NaB. Scale bars = 100 μm. *P < 0.05, **P < 0.01, One-way

analysis of variance (ANOVA).

sFig. 4 The silence efficiency of HDAC9

a The expression of *HDAC9* mRNA were examined by qRT-PCR in aged BMMSCs after transfected with an *HDAC9* siRNA for 48 hours. b, c Protein level of HDAC9 (b) and acetylation of H3K9 (c) were examined by western blotting in aged BMMSCs after transfection with an *HDAC9* siRNA for 48 hours. d HDAC9 mRNA expression was measured by qRT-PCR 3 days and 7 days after siHDAC9 transfection in aged BMMSCs. e HDAC9 protein expression was measured by western blotting 3 days and 7 days after siHDAC9 transfection in aged BMMSCs. The data are presented as the means ± s.d. of each independent experiment performed in triplicate. *$P < 0.05$, **$P < 0.01$. a unpaired two-tailed Student's t-test. d One-way analysis of variance (ANOVA).

sFig. 5 *HDAC9* siRNA restored the number of autophagosomes in aged BMMSCs

a Transmission electron microscopy (TEM) was used to detect autophagosomes in young and aged BMMSCs and cells treated with chloroquine (CQ). Scale bars = 1 μm. b Transmission electron microscopy (TEM) was used to detect autophagosomes in aged BMMSCs respectively transfected with Nc siRNA, HDAC9 siRNA and cells treated with CQ, respectively. Scale bars = 1 μm. The data are presented as the means ± s.d. of each independent experiment performed in triplicate. *$P < 0.05$, ** $P < 0.01$, *** $P < 0.001$, One-way analysis of variance (ANOVA).

sFig. 6 HDAC9 siRNA treatment decreased the levels of acetylation of H3K9 in aged BMMSCs cultured *in vitro*

To evaluate the effect of HDAC9 removing acetyl groups from H3K9, the expression of HDAC9 and the levels of acetylation of H3K9 were examined by western blotting in aged BMMSCs transfected with an *HDAC9* siRNA and those cells treated with CQ. The data are presented as the

means ± s.d. of each independent experiment performed in triplicate.

sFig. 7 The silence efficiency of BECN1 in aged BMMSCs cultured in vitro

Protein expression of Beclin1 was examined by western blotting in aged BMMSCs transfected with a *BECN1* siRNA 48 hours later. The data are presented as the means ± s.d. of each independent experiment performed in triplicate.

sFig. 8 The thickness of growth plate in femora were no changes in aged mice treatment with HDAC9 shRNA lentivirus

The chondrocytes in growth plate of femur were recognized with Alcian blue staining and thickness of growth plate was quantitative analyzed. The data are presented as the means ± s.d. of each independent experiment performed in triplicate. One-way analysis of variance (ANOVA).

Supplementary Figure 1

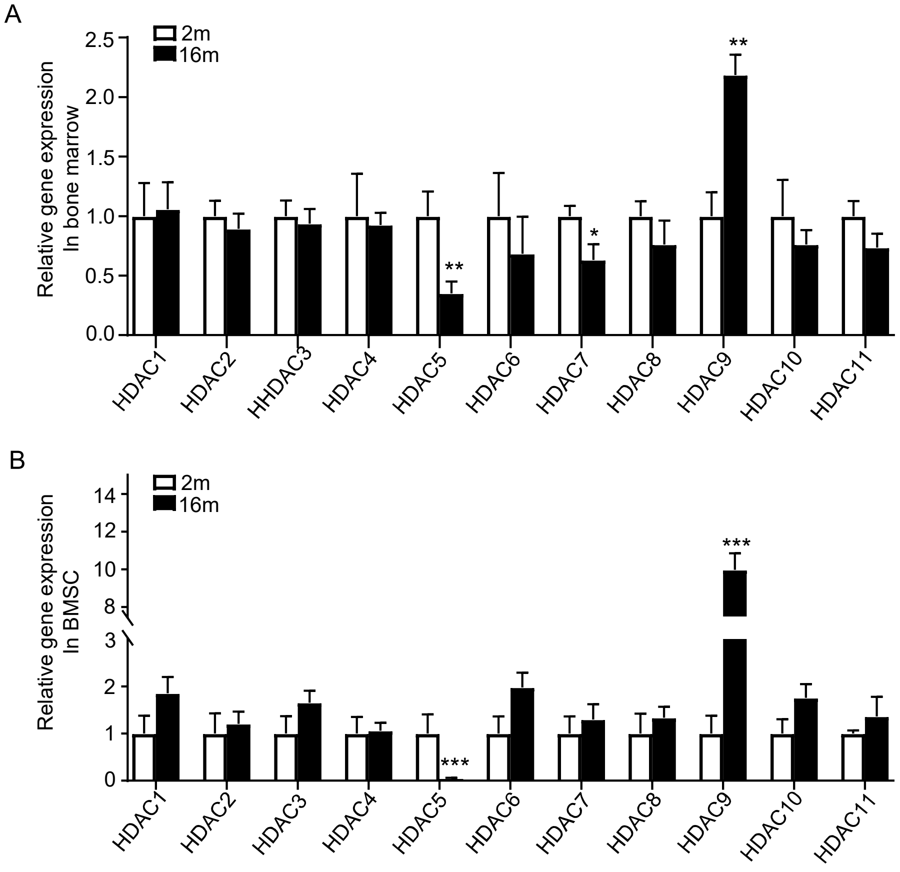

Supplementary Figure 2

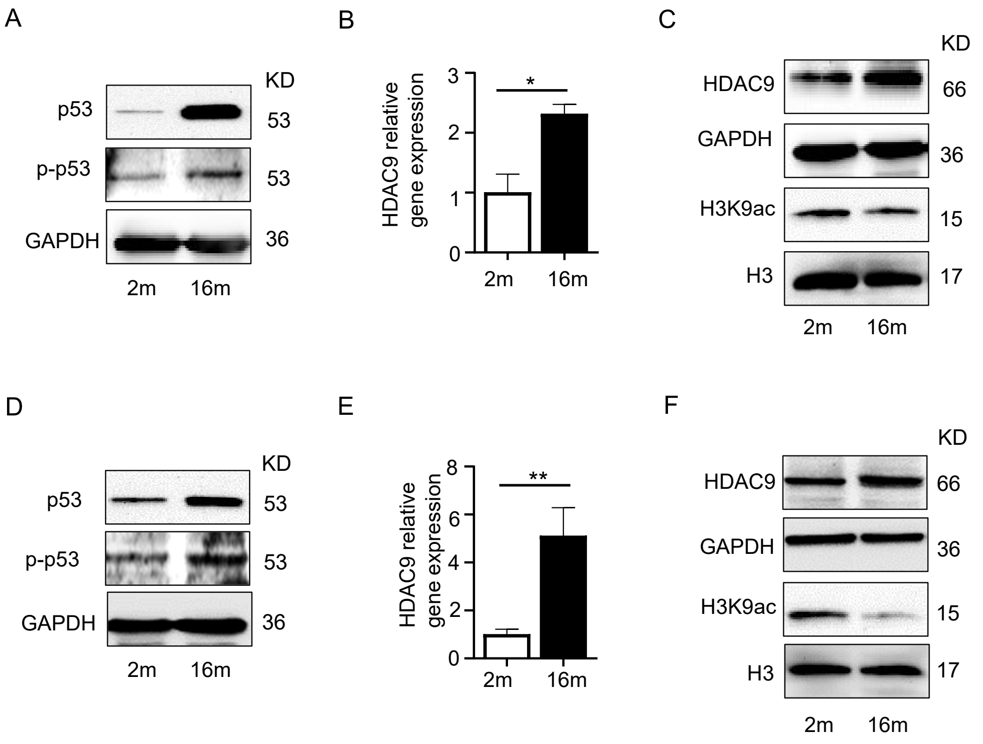

Supplementary Figure 3

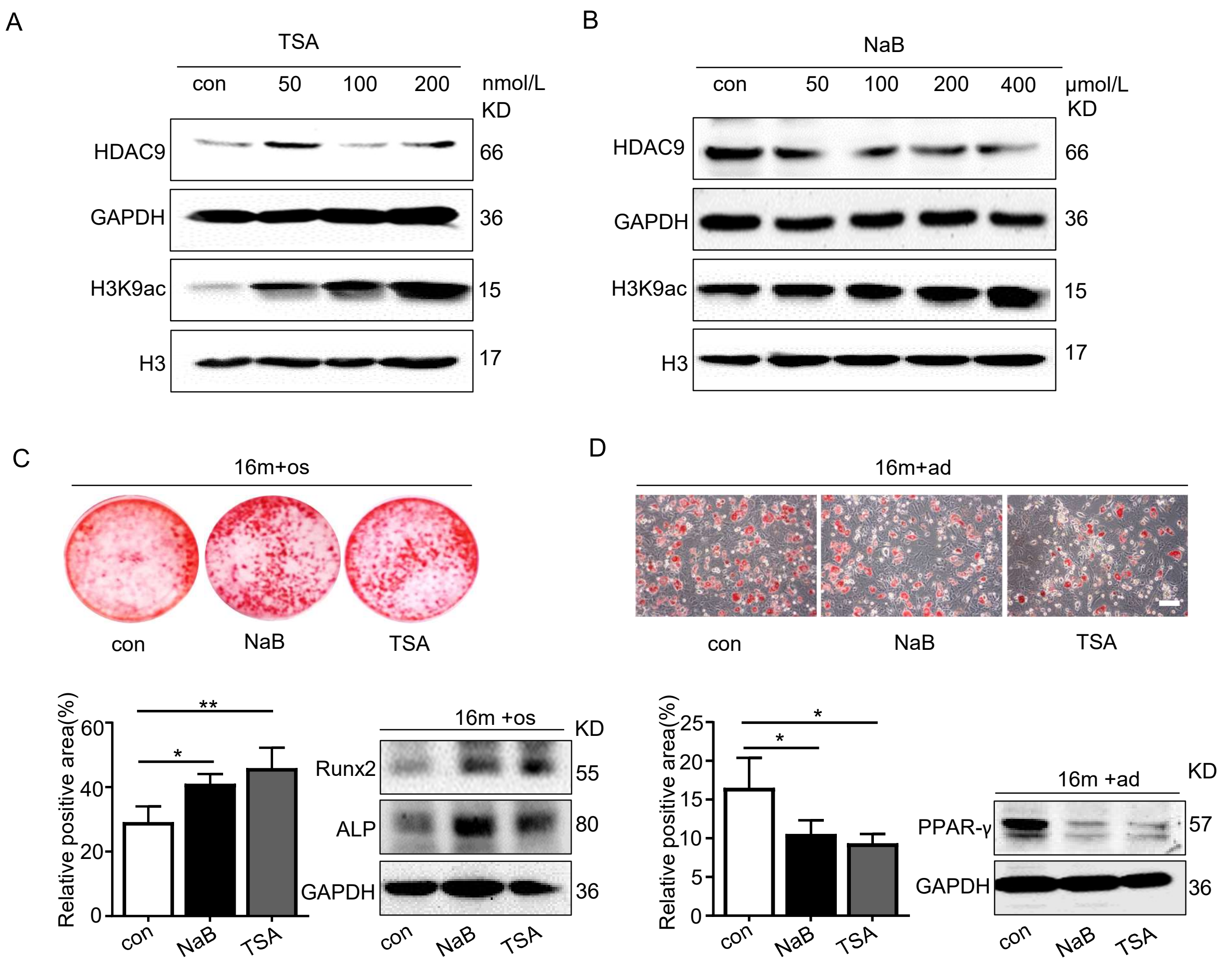

Supplementary Figure 4

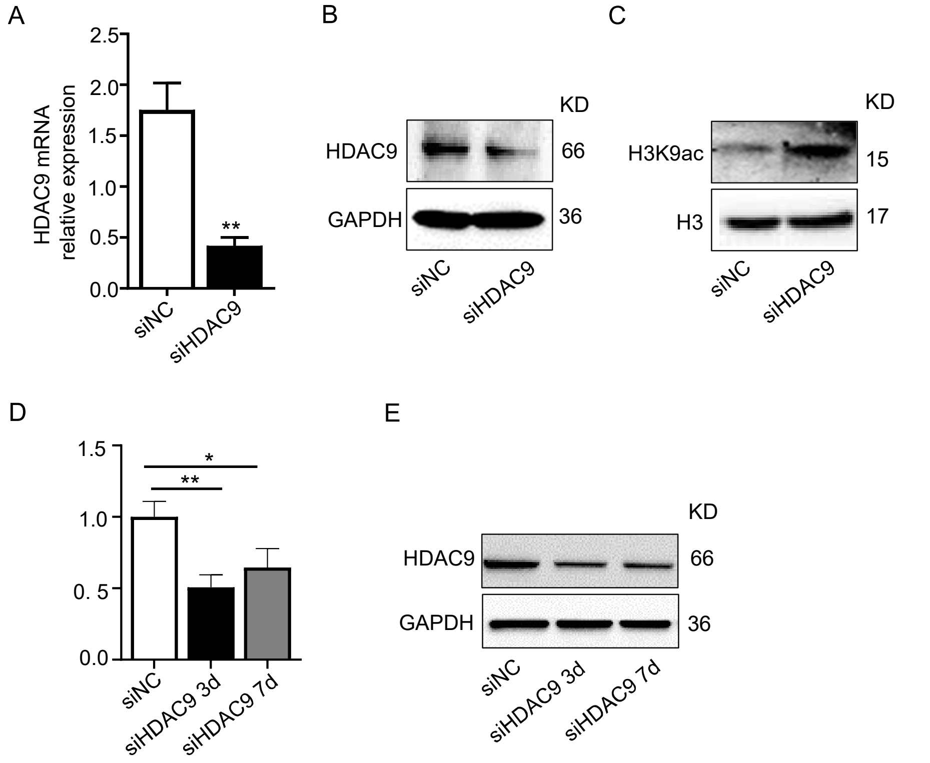

Supplementary Figure 5

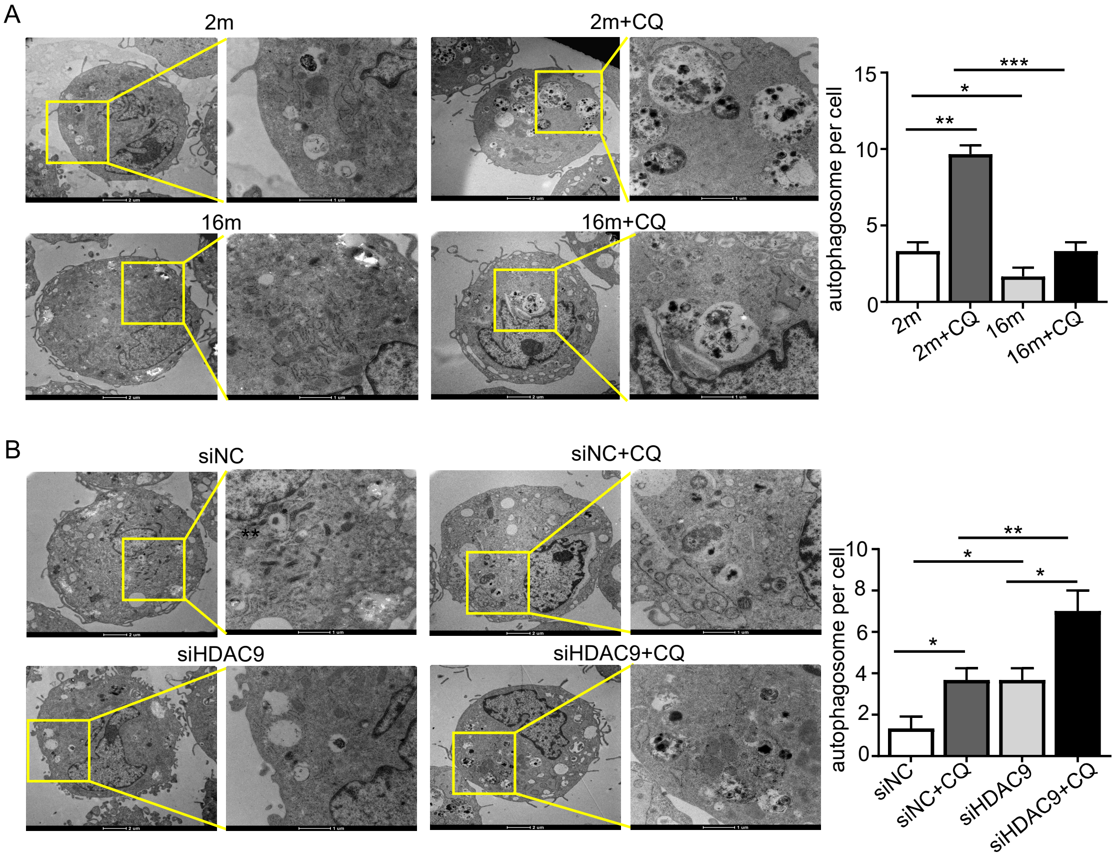

Supplementary Figure 6

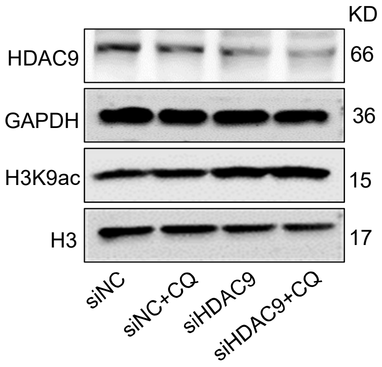

Supplementary Figure 7

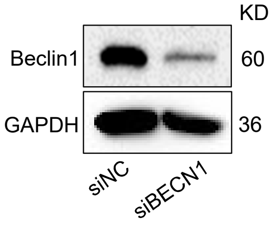

Supplementary Figure 8

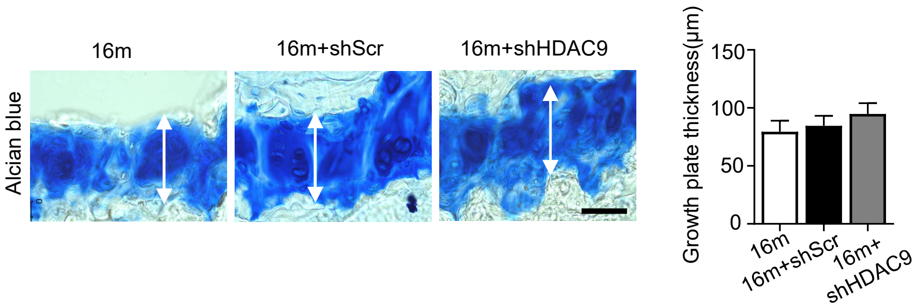

Supplement: Supplementary file 1 — Additional file 1: sFig. 1 The expressions of HDACs family members in bone marrow and BMMSCs HDACs gene were examined in bone marrow (a) and BMMSCs (b) from young mice and aged mice by qRT-PCR. sFig. 2 The expressions of senescence associated proteins and HDAC9 in skeletal muscle and myoblasts. The expression of p53, HDAC9 and H3K9 acetylation, and HDAC9 in skeletal muscle (a-c) and myoblasts (d-f) were examined by western blotting and qRT-PCR from young mice and aged mice. sFig. 3 HDACs inhibitors promoted osteogenic differentiation and inhibited adipogenic differentiation in aged BMMSCs The expression of HDAC9 and H3K9 acetylation were detected in aged BMMSCs treatment with different dose of TSA (a) or NaB (b) by western blotting. Osteogenic differentiation (c) and adipogenic differentiation (d) were observed in aged BMMSCs treated with TSA or NaB. Scale bars = 100 μm. sFig. 4 The silence efficiency of HDAC9 siRNA HDAC9 and H3K9 acetylation were examined in aged BMMSCs after transfected with HDAC9 siRNA for 48 hours, 3 days and 7days by qRT-PCR (a, d) and western blotting (b, c, e). sFig. 5HDAC9 siRNA restored the number of autophagosomes in aged BMMSCs Autophagosomes in young, aged BMMSCs (a) and aged BMMSCs transfected with HDAC9 siRNA (b) were detected by TEM. Scale bars = 1 μm. sFig. 6HDAC9 siRNA decreased H3K9 acetylation in aged BMMSCs. The expression of HDAC9 and H3K9 acetylation were examined by western blotting in aged BMMSCs transfected with HDAC9 siRNA and those cells treated with CQ. sFig. 7 The silence efficiency of BECN1 siRNA Beclin1 was examined by western blotting in aged BMMSCs transfected with BECN1 siRNA 48 hours later. sFig. 8 No changes in growth plate thickness in aged mice were treated with HDAC9 shRNA lentivirus Alcian blue staining were performed to assess growth plate thickness. [file 13287_2020_1785_MOESM1_ESM.pdf]
