# Supplementary material for: Impaired autophagy triggered by HDAC9 in mesenchymal stem cells accelerates bone mass loss
Source: Stem Cell Res Ther. 2020 Jul 3;11:269. doi: 10.1186/s13287-020-01785-6 (PMC7333327; doi:10.1186/s13287-020-01785-6)
Supplement: Supplementary file 2 — Additional file 2: Supplementary Table 1. Primer sequences for Real-Time PCR assay. Supplementary Table 2. Primer sequences for ChIP assay. [file 13287_2020_1785_MOESM2_ESM.docx]

**Supplementary Tables**

Supplementary Table 1

Primer sequences for Real-Time PCR assay

| Gene name | Forward primer | Reverse primer |
| --- | --- | --- |
| *GAPDH* | TGTGTCCGTCGTGGATCTGA | TTGCTGTTGAAGTCGCAGGAG |
| *HADC1* | CCCATGAAGCCTCACCGAAT | CAAACACCGGACAGTCCTCA |
| *HADC2* | TATCCCGCTCTGTGCCCTAC | GAGGCTTCATGGGATGACCC |
| *HADC3* | FCCCCACCAATATGCAGGGTT | CAGAAGCCAGAGGCCTCAAA |
| *HADC4* | GGGAGCAGCATCATGGTTCA | CTAGCAGCGTCAGTGCCTTA |
| *HADC5* | CCGGGAACCATCCTTGGAA | GGGCTACCTCCACCTCCA |
| *HADC6* | CACCGCATTCAGAGGGTTCT | CCTTAAGGTGGGGCCAGAAG |
| *HADC7* | TCCGTGCTGATACCTCTGGC | TCGGGATGCTTGCTGTTGT |
| *HADC8* | CTGCACAAGTTCTCCCCAGG | AGGAAGGGAGGTGTTTCTGGA |
| *HADC9* | GCCTCAGAGCCCAACTTGAA | TTCGGTCACATTCCCAGCAG |
| *HADC10* | GCTTCACAAATCCCGGTTCC | AGCCACAGAATTCTCCCATCA |
| *HADC11* | CTGGCCCATCGTGTACTCAC | GTTGAGATAGCGCCTCGTGT |
| *BECN1* | CAGTACCAGCGGGAGTATAGTGA | TGTGGAAGGTGGCATTGAAGA |
| *LC3* | CCTGTCCTGGATAAGACCAAGTT | CTCCTGTTCATAGATGTCAGCGAT |
| *Atg7* | GGTCGTGTCTGTCAAGTGC | CTCCCTGGTGTCCATTAGC |
| *p62* | CTCTCATAGCCGCTGGCTTC | CCTCAATGCCTAGAGGGCTG |

Supplementary Table 2

Primer sequences for ChIP assay

| Gene name | Forward primer | Reverse primer |
| --- | --- | --- |
| *Atg7* | GCAAAGCAAAGGTAAGCAAT | GCTGTTCTGGACCTGTATAC |
| *BECN1* | AACAAACAAACAACACCCTG | TAGACGGTCCACTCAAGATT |
| *LC3a* | TTTCAGAGGACCTAAGCTTG | AACATAGCATCGGATTTCCT |
| *LC3b* | TCTCCAGACGTCTCCATAAT | TCCTAAATTCTACCCACCCC |
